# HELLPAR/RRM2 axis related to HMMR as novel prognostic biomarker in gliomas

**DOI:** 10.1186/s12885-023-10596-w

**Published:** 2023-02-07

**Authors:** Huaxin Zhu, Jiacong Tan, Xinyi Pan, Hengyang Ouyang, Zhixiong Zhang, Meihua Li, Yeyu Zhao

**Affiliations:** 1 Department of Neurosurgery, the First Affiliated Hospital of Nanchang University, No. 17 Yongwaizheng Street, Nanchang, 330006 Jiangxi China; 2 Huankui Academy, Nanchang University, Honggutan New District, Jiangxi 330006 Nanchang, China

**Keywords:** HMMR, HELLPAR/RRM2 axis, Gliomas, Prognosis, ceRNA network

## Abstract

**Background:**

Gliomas are the most frequent type of central nervous system tumor, accounting for more than 70% of all malignant CNS tumors. Recent research suggests that the hyaluronan-mediated motility receptor (HMMR) could be a novel potential tumor prognostic marker. Furthermore, mounting data has highlighted the important role of ceRNA regulatory networks in a variety of human malignancies. The complexity and behavioural characteristics of HMMR and the ceRNA network in gliomas, on the other hand, remained unknown.

**Methods:**

Transcriptomic expression data were collected from TCGA, GTEx, GEO, and CGGA database.The relationship between clinical variables and HMMR was analyzed with the univariate and multivariate Cox regression. Kaplan–Meier method was used to assess OS. TCGA data are analyzed and processed, and the correlation results obtained were used to perform GO, GSEA, and ssGSEA. Potentially interacting miRNAs and lncRNAs were predicted by miRWalk and StarBase.

**Results:**

HMMR was substantially expressed in gliomas tissues compared to normal tissues. Multivariate analysis revealed that high HMMR expression was an independent predictive predictor of OS in TCGA and CGGA. Functional enrichment analysis found that HMMR expression was associated with nuclear division and cell cycle. Base on ssGSEA analysis, The levels of HMMR expression in various types of immune cells differed significantly. Bioinformatics investigation revealed the HEELPAR-hsa-let-7i-5p-RRM2 ceRNA network, which was linked to gliomas prognosis. And through multiple analysis, the good predictive performance of HELLPAR/RRM2 axis for gliomas patients was confirmed.

**Conclusion:**

This study provides multi-layered and multifaceted evidence for the importance of HMMR and establishes a HMMR-related ceRNA (HEELPAR-hsa-let-7i-5p-RRM2) overexpressed network related to the prognosis of gliomas.

**Supplementary Information:**

The online version contains supplementary material available at 10.1186/s12885-023-10596-w.

## Introduction

Gliomas are the most frequent type of central nervous system(CNS) tumor, accounting for more than 70% of all malignant CNS tumors, with an annual incidence rate of 6.6 per 100,000 population [1–3]. Unfortunately, half of newly diagnosed gliomas are the most malignant glioblastoma, with median patient survival duration of approximately 14 to 17 months [4–7]. Low-grade gliomas have the potential to evolve into more aggressive and glioblastoma (GBM) in spite of its encouraging prognosis [8, 9]. Over the last decade isocitrate dehydrogenase (IDH) mutation, chromosome 1p/19q deletion, MGMT promoter methylation, TERT promoter mutation and histone mutation have been identified as biomarkers and play a central role for classification of gliomas and treatment decisions [10–12]. However, the molecular understanding of gliomas is still limited, the treatment of gliomas is full of challenges and prognosis is not optimistic. Therefore, it is important to find new biomarkers to provide a highly reliable prediction of a patient,s survival and more aggressive treatment.

The cell membrane receptor hyaluronan-mediated motility receptor (HMMR), which is linked to the Glycosaminoglycan hyaluronic acid (HA), is significantly expressed in a variety of malignant tumors, including breast cancer, stomach carcinoma, bladder cancer, prostate carcinoma, colorectal cancer, and others [13–17]. According to several research, increased HMMR expression is linked to a bad prognosis because it speeds up tumor growth and metastasis. However, for malignant peripheral nerve sheath tumor and other tumors, poor patient survival associates with low HMMR expression [18, 19]. Although HMMR was shown to be overexpressed in GBM stem cells and might be used as a biomarker for gliomas, its predictive usefulness and putative function in gliomas were unknown and unproven [20].

Noncoding RNAs, including long noncoding RNAs (lncRNAs), short microRNAs (miRNAs), and circular RNAs (circRNAs), account for 95% of total eukaryotic transcripts [21]. LncRNAs are ncRNA subtypes with a length more than 200 nt and no or limited ability to code for proteins [22]. LncRNAs have been shown to play both direct and indirect regulatory roles in cancer biology in a number of studies [23–25]. Specific lncRNAs have been linked to cancer recurrence, metastasis, and poor prognosis in several cancer types, including gliomas [26–28]. During the disease process of gliomas, lncRNA LINC01057 can promote mesenchymal differentiation by activating NF-kB signaling in glioblastoma, lncRNA BCYRN1 can prevent gliomas carcinogenesis by competitively interacting with miR-619-5p to modulate CUEDC2 expression, lncRNA PVT1 can enhance gliomas tumorigenesis and progression by modulating the MiR-128-3p/GREM1 axis and the BMP signaling pathway and the like [29–31].

MicroRNAs (miRNAs) are short single-stranded noncoding RNAs (ncRNAs) with 19–25 nucleotides that can attach to target mRNAs and prevent gene destruction or translation [32–34]. MiRNAs also control roughly 30% of the genes in the human genome [35, 36]. At present, it is mainly believed that in the cytoplasm, lncRNAs regulate mRNA by adsorbing miRNAs through a competitive endogenous RNA (ceRNA) regulatory mechanism [37, 38]. The lncRNA KTN1-AS1 negatively regulates miR-505-3p through ceRNA action to promote glioma cell proliferation and invasion [39]. LncRNA NEAT1 and lncRNA NFIA-AS2 can promote gliomas progression by regulating miR-98-5p/BZW1 and miR-655-3p/ZFX axis, respectively [40, 41]. These findings suggest that the ceRNA network plays an important role in the disease progression of gliomas.

**Fig. 1:**
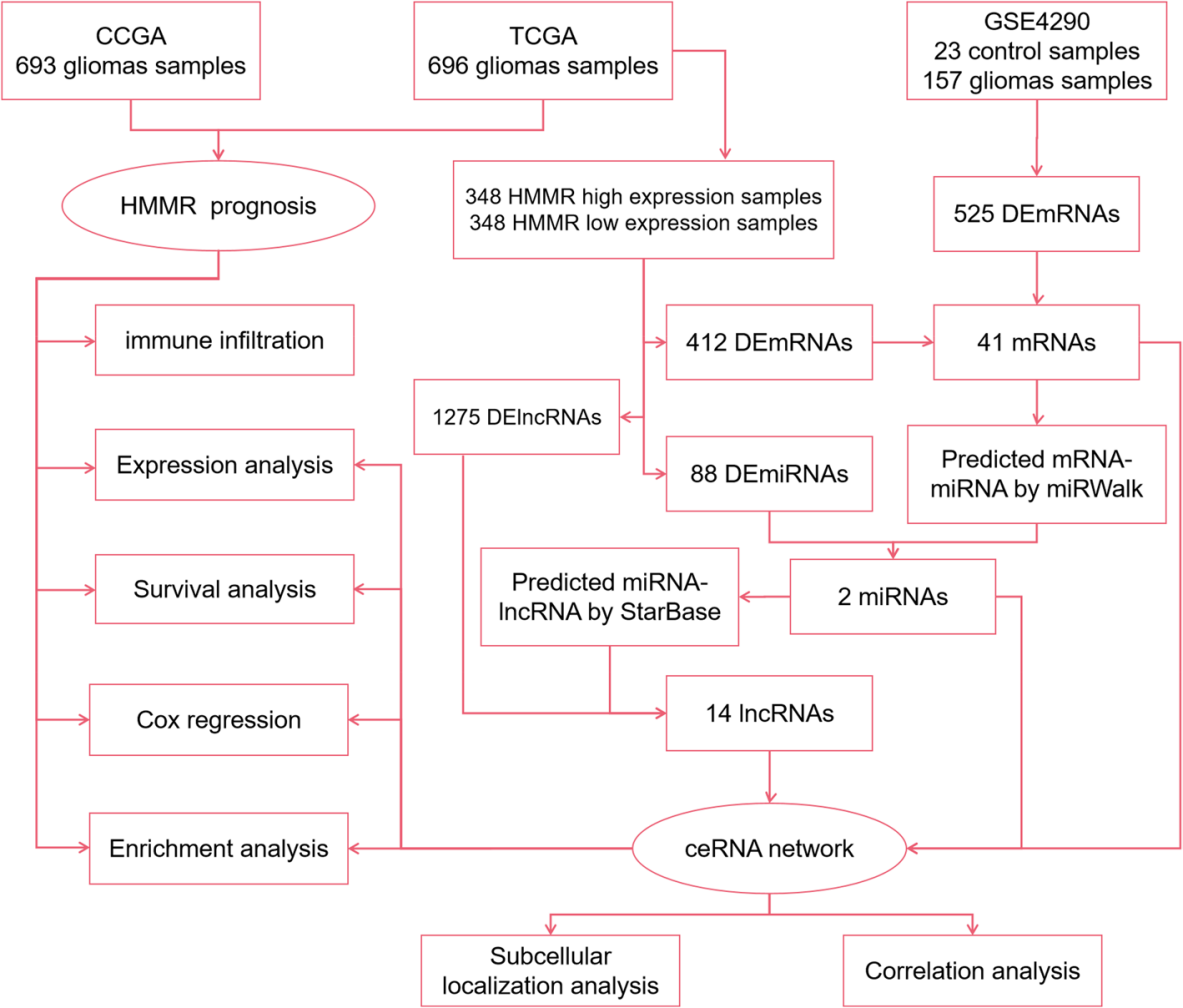
Flowchart of analysis of HMMR and construction of ceRNA

In this study, we comprehensively evaluated the predictive value of HMMR and constructed a HMMR related ceRNA network in patients with gliomas using data from the Cancer Genome Atlas (TCGA) database, Chinese Gliomas Genome Atlas (CGGA) databases and Gene Expression Omnibus (GEO) database (Fig. 1). First, we used expression analysis, survival analysis, cox regression analysis to demonstrate that the level of HMMR expression was found to be substantially linked to the poor prognosis of glioma patients. Based on the high and low HMMR expression we analyzed the differentially expressed mRNA (DEmRNAs) from TCGA for GO, KEGG, and GSEA enrichment analysis. Then, these DEmRNAs were also jointly analyzed with the DEmRNAs from GSE4290 to construct a lncRNA-miRNA-mRNA triple regulatory network. Furthermore, survival analysis, nuclear-cytoplasmic localization study, and correlation analysis of RNAs from triple regulatory networks showed a critical ceRNA network (HELLPAR-hsa-let-7i-5p-RRM2).Finally, the diagnostic and prognostic values of RRM2 in gliomas were determined using expression analysis, survival analysis, and cox regression analysis.

## Materials and methods

### Data acquisition

The gliomas RNA-seq data of TCGA and corresponding normal tissue data of GTEx by toil processing uniformly were downloaded from UCSCXENA(https://xenabrowser.net/datapages/). According to the median HMMR expression value, patients with gliomas were divided into two groups: low-expression and high-expression. The gene expression profiles of GSE4290 were extracted from the GEO database (https://www.ncbi.nlm.nih.gov/geo/). The Human Protein Atlas (https://www.proteinatlas.org/) database was used to examine HMMR protein expression in normal and RRM2 protein expression in normal and gliomas tissues. The study also downloaded glioma samples RNA-seq data and clinical information (DataSet ID: mRNAseq_693) of the CGGA (http://www.cgga.org.cn/) as an external validation of survival analyses.

### Analysis of differentially expressed mRNAs (DEmRNAs), differentially expressed miRNAs (DEmiRNAs), and differentially expressed lncRNAs (DElncRNAs) between the high and low HMMR expression groups in patients with gliomas

In this study, R package DESeq2 (1.26.0) was used to analyze between low and high HMMR mRNA expression for obtaining DEmRNAs, DEmiRNAs, and DElncRNAs [42]. The limma (version 3.48.1) R package was used to filter the DEmRNAs between the gliomas and control samples from GSE4290 [43]. |log2FC|> 2.0 and adjusted *P*-value < 0.05 were considered the threshold for the DEmRNAs. |log2FC|> 0.5 and adjusted *P*-value < 0.05 were considered the threshold for the DEmiRNAs. |log2FC|> 1.0 and adjusted *P*-value < 0.05 were considered the threshold for the DElncRNAs.

### Gene ontology (GO) and Kyoto Encyclopedia of Genes and Genomes (KEGG) enrichment analysis and gene set enrichment analysis (GSEA)

The R package clusterProfiler package (3.14.3) is used for GO and KEGG enrichment analysis. GSEA was carried out using the R package clusterProfiler, which performed 1000 times of gene set permutations for each analysis [44–46]. We chose c2.cp.v7.2.symbols.gmt as the reference gene collection in the MSigDB Collections. An adjusted *P*-value < 0.05, False discovery rate (FDR) < 0.05 and normalized enrichment score (NES) > 1 were considered as significant enrichment.

### HMMR expression is correlated with immune infiltration level in gliomas

We quantified 24 types of immune cells associated with levels of glioma immune infiltration to evaluate the correlation between immune cells and HHMR expression by applying the ssGSEA (single-sample Gene Set Enrichment Analysis) method from the GSVA package (1.34.0) in R [47]. The link between distinct HMMR mRNA expression levels and immune cell infiltration in gliomas samples from the TCGA database was validated using the TIMER software.

### Prognostic model construction and external validation

On the basis of the TCGA database, univariate and multivariate Cox regression analyses of clinical variables were done to screen for relevant prognostic factors, which were then utilized to build the best prognostic model. R packages rms were used to create a nomogram that predicted prognosis. Kaplan–Meier curve was constructed to show the difference between two groups in overall survival(OS), disease specific survival(DSS) and progress free interval(PFI). Harrell,s concordance index (C-index), calibration plots were formulated to evaluate the reliability and accuracy of the prognostic model intensity.Receiver operating characteristic (ROC) curve was performed to compare the prediction accuracy. The CGGA database was used for external validation to verify relevant results.

### Gene-miRNA and miRNA-lncRNA interaction networks

We used miRWalk (version 3; http://mirwalk.umm.uni-heidelberg.de/) to predict targeted pivotal miRNAs and build the DEmRNAs-miRNA interaction networks. We intersected the predicted results of the TargetScan and miRWalk database to ensure the accuracy of the results. Upstream lncRNAs of key miRNAs were identified by using StarBase (https://starbase.sysu.edu.cn/) [48]. The target sites were predicted by StarBase. The DElncRNA sequences were obtained from LNCipedia (https://lncipedia.org/), and the lncLocator (http://www.csbio.sjtu.edu.cn/bioinf/ lncLocator/) database was used to determine the DElncRNA's cellular localization based on its sequence. Networks were visualized in Cytoscape software (version 3.8.2).

### Immunohistochemistry (IHC)

Paraffin-embedded human gliomas tissue samples were obtained from the Department of Pathology, The First Affiliated Hospital of Nanchang University. The Ethics Committee of the First Affiliated Hospital of Nanchang University approved this study. Paraffin tissues were dewaxed and hydrated with xylene and ethanol. The sections were immersed in citrate retrieval solution for antigen retrieval and then heated in a microwave oven. Then sections were washed with distilled water and incubated in a 3% aqueous hydrogen peroxide solution and blocked with blocking solution for 1 h at room temperature. Add HMMR antibody [ab124729](1:100)(Abcam, UK) and incubate overnight at 4 °C. After the antibody reaction was completed, sections were incubated with secondary antibodies, 3,3' diaminobenzidine (DAB) solution was used as chromogen and Harris hematoxylin was used as counterstain. Finally, the slides were dehydrated and mounted. Images were captured using a Leica microscope. Image-J software (version 6.0, Rockville, MD, USA) was used to evaluate the area and integrated optical density (IOD) values of the stained areas of the IHC sections.

### Statistical analysis

This study analyzed the expression of HMMR in non-paired and paired samples by applying Shapiro–Wilk normality test and Wilcoxon rank-sum. The Dunn, s test, Kruskal–Wallis Test and Shapiro–Wilk normality test were used to analyze the correlations between the clinical characteristics and HMMR and RRM2 expression. OS, DSS and PFI curves were established by using the Kaplan–Meier method between the two groups via the log-rank test. Univariate and multivariate Cox regression analyses of clinical variables were performed to screen for significant prognostic factors to build a nomogram. Statistical analyses were carried out using R (version 3.6.3).

## Results

### The expression of HMMR in gliomas

**Fig. 2:**
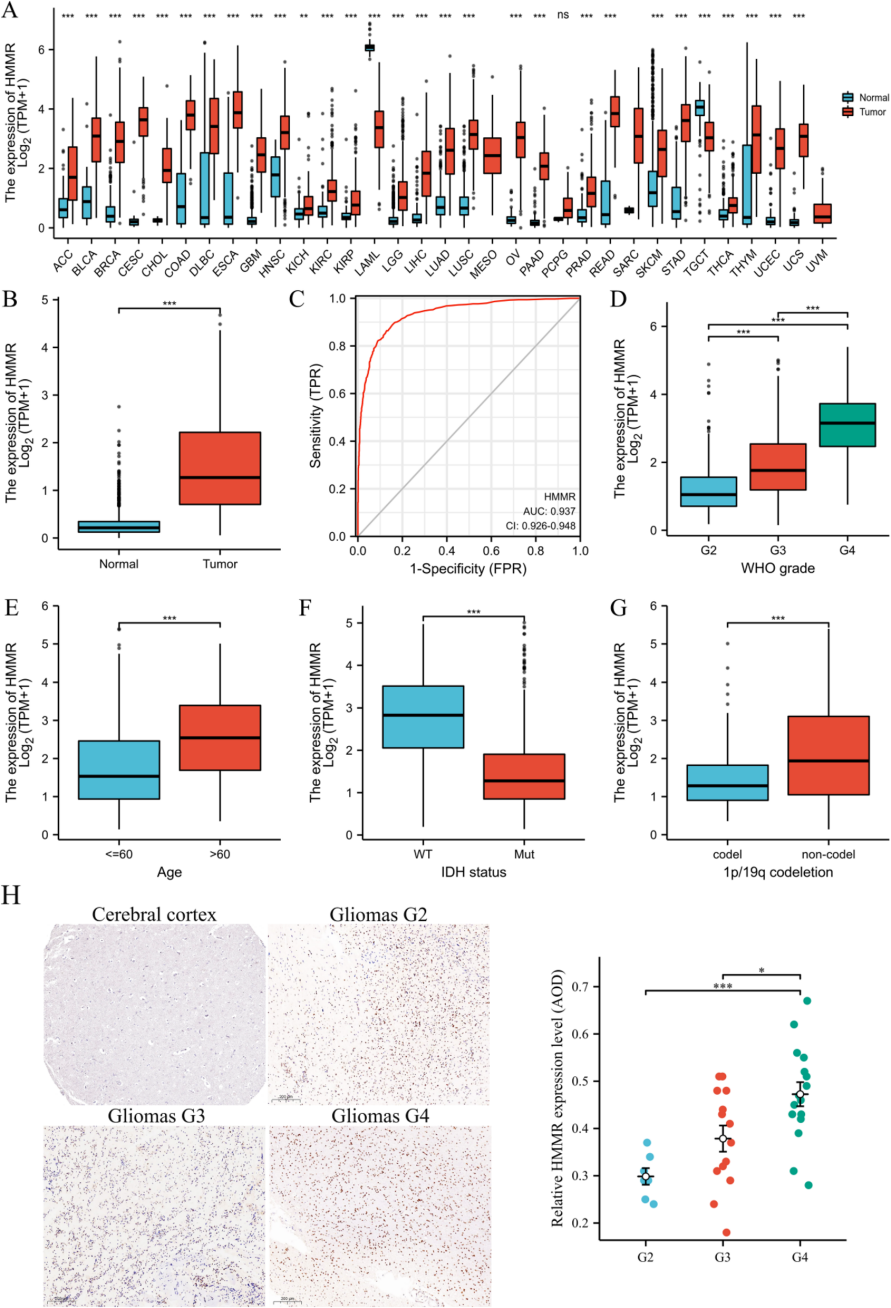
Expression patterns of HMMR mRNA in gliomas. **A** HMMR expression levels in different tumor types from TCGA database. **B** HMMR mRNA expression was significantly upregulated in gliomas tissues compared to normal tissues. **C** Diagnostic value of HMMR expression in gliomas. **D-G** The association of HMMR expression with WHO grade (**D**), age (**E**), IDH status (**F**), and 1p/19q codeletion (**G**) in gliomas from TCGA database. **H** HMMR protein expression in gliomas tissues determined (cerebral cortex image was obtained from HPA).*p < 0.05, **p < 0.01, ***p < 0.001

Firstly, We found that HMMR was highly expressed in gliomas by the analysis of the pan-cancer RNA-seq data from TCGA (Fig. 2A). To further study the expression of HMMR, we compared the expression level of HHMR in glioma and normal tissues The expression level of HHMR in glioma was significantly higher than that in normal tissues (*p* < 0.01; Fig. 2B). Receiver operating characteristic curve (ROC) analysis was applied to evaluate the effectiveness of HMMR mRNA expression level to distinguish gliomas from normal tissues, which estimated AUC at 0.937 (95% CI: 0.926–0.948; Fig. 2C). It is well known that prognosis of glioma is closely related to WHO grading, IDH, age, and chromosome 1p/19q. High HMMR expression was closely associated with high grade of WHO (Fig. 2D), age > 60 years (Fig. 2E), IDH wild type (Fig. 2F) and intact 1p /19q (Fig. 2G). We also found that immunohistochemical analysis of HMMR was positive in gliomas and significantly highly expressed in grade 4 (G4), while negative in normal tissues (Fig. 2H).

### The prognostic significance of HMMR in gliomas patients

**Fig. 3:**
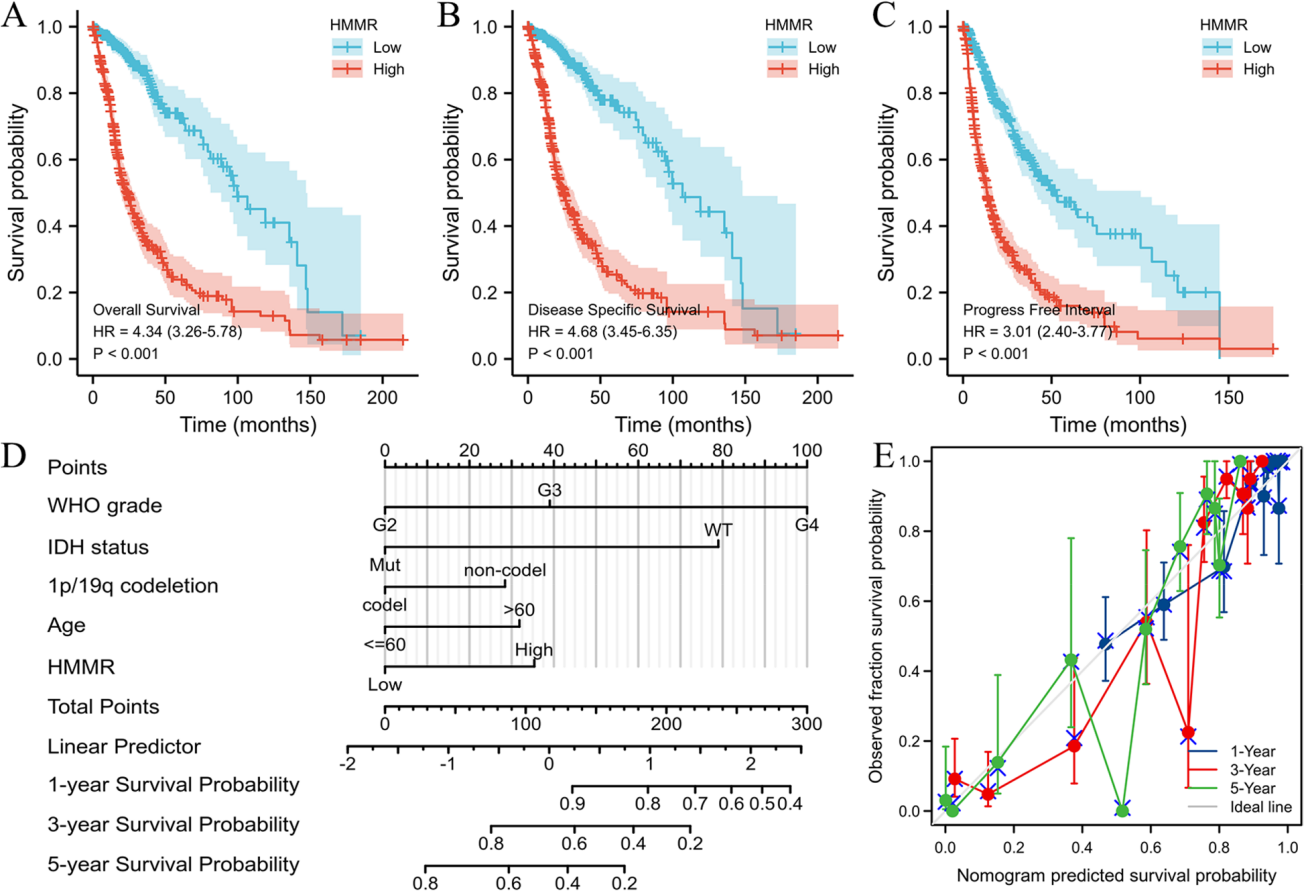
High expression of HMMR indicates poor survival in patients with gliomas from TCGA database. **A-C** Kaplan–Meier survival curve analysis of overall survival(OS) (**A**), disease specific survival(DSS) (**B**) and progress free interval(PFI) (**C**) showed that high HMMR expression correlated to poor prognosis of gliomas patients from TCGA database. **D** A nomogram that integrates HMMR and other prognostic factors in gliomas from TCGA database. **E** The calibration curve of the nomogram

In this study, clinical information of 696 glioma patients was obtained from TCGA database, including age, gender, WHO grade, IDH status, 1p/19q codeletion, overall survival (OS) event, disease specific survival (DSS) event, progress free interval (PFI) event (Table 1). We analyzed clinical variables by univariate and multivariate COX regression (Table 2). Compared with HMMR low expression group, HMMR high expression group had worse OS (HR = 4.34(3.26–5.78), log-rank *P* < 0.001), DSS (HR = 4.68(3.45–6.35), log-rank *P* < 0.001) and PFI (HR = 3.01(2.40–3.77), log-rank *P* < 0.001) by the Kaplan–Meier method with a two-sided log-rank test (Fig. 3A-C). A nomogram of OS was established based on HHMER and other clinical variables (age, WHO grade, IDH status, 1p/19q codeletion) (Fig. 3D). Harrell’s concordance index (C-index = 0.850(0.839–0.861)) and a calibration curve (Fig. 3E) were formulated to evaluate the discrimination and calibration of the nomogram.

**Table 1:** Association of HMMR expression and demographic and clinical characteristics in patients with gliomas

Characteristic	TCGA			CGGA		
	Low	High	*p*	Low	High	*p*
n	348	348		346	347	
Gender, n (%)			0.592			0.668
Female	153 (22%)	145 (20.8%)		144 (20.8%)	151 (21.8%)	
Male	195 (28%)	203 (29.2%)		202 (29.1%)	196 (28.3%)	
Age, meidan (IQR)	40 (32, 51)	53 (38, 63)	** < 0.001**	42 (34, 50.75)	43 (34.25, 53)	0.246
Age, n (%)			** < 0.001**			0.21
< = 60	312 (44.8%)	241 (34.6%)		316 (45.7%)	305 (44.1%)	
> 60	36 (5.2%)	107 (15.4%)		30 (4.3%)	41 (5.9%)	
WHO grade, n (%)			** < 0.001**			** < 0.001**
G2	182 (28.7%)	42 (6.6%)		129 (18.6%)	59 (8.5%)	
G3	115 (18.1%)	128 (20.2%)		132 (19.1%)	123 (17.8%)	
G4	9 (1.4%)	159 (25%)		85 (12.3%)	164 (23.7%)	
IDH status, n (%)			** < 0.001**			**0.006**
WT	45 (6.6%)	201 (29.3%)		114 (17.8%)	172 (26.8%)	
Mut	300 (43.7%)	140 (20.4%)		182 (28.3%)	174 (27.1%)	
1p/19q codeletion, n (%)			** < 0.001**			**0.134**
codel	121 (17.6%)	50 (7.3%)		74 (11.9%)	71 (11.4%)	
non-codel	226 (32.8%)	292 (42.4%)		208 (33.4%)	270 (43.3%)	
OS event, n (%)			** < 0.001**			** < 0.001**
Alive	287 (41.2%)	137 (19.7%)		175 (26.4%)	91 (13.7%)	
Dead	61 (8.8%)	211 (30.3%)		156 (23.5%)	241 (36.3%)	
DSS event, n (%)			** < 0.001**			
Alive	288 (42.7%)	143 (21.2%)				
Dead	53 (7.9%)	191 (28.3%)				
PFI event, n (%)			** < 0.001**			
Alive	237 (34.1%)	113 (16.2%)				
Dead	111 (15.9%)	235 (33.8%)				

*OS* Overall survival, *DSS* Disease specific survival, *PFI* Progress free interval

**Table 2:** Univariate and multivariate analysis of demographic and clinical characteristics that correlate with OS of gliomas patients (TCGA database)

Characteristics	Total(N)	Univariate analysis		Multivariate analysis	
		Hazard ratio (95% CI)	*P* value	Hazard ratio (95% CI)	*P* value
WHO grade	634				
G2	223	Reference			
G3	243	2.999 (2.007–4.480)	** < 0.001**	1.694 (1.088–2.636)	**0.020**
G4	168	18.615 (12.460–27.812)	** < 0.001**	3.802 (2.200–6.569)	** < 0.001**
1p/19q codeletion	688				
codel	170	Reference			
non-codel	518	4.428 (2.885–6.799)	** < 0.001**	1.486 (0.900–2.455)	0.122
Age	695				
< = 60	552	Reference			
> 60	143	4.668 (3.598–6.056)	** < 0.001**	1.530 (1.124–2.081)	**0.007**
Gender	695				
Female	297	Reference			
Male	398	1.262 (0.988–1.610)	0.062	1.213 (0.923–1.595)	0.166
IDH status	685				
WT	246	Reference			
Mut	439	0.117 (0.090–0.152)	** < 0.001**	0.314 (0.209–0.473)	** < 0.001**
HMMR	695				
Low	348	Reference			
High	347	4.344 (3.264–5.780)	** < 0.001**	1.650 (1.147–2.372)	**0.007**

**Fig. 4:**
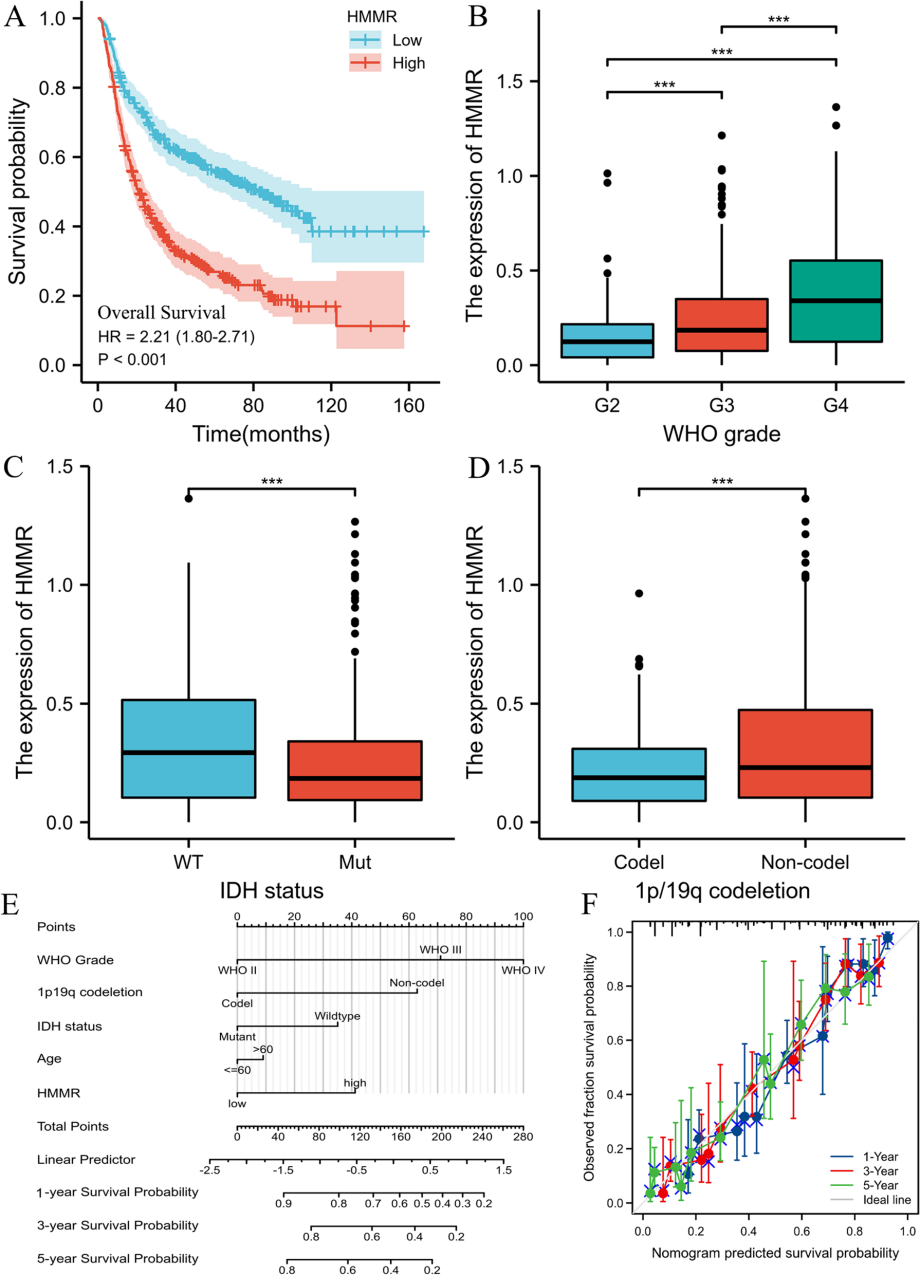
High expression of HMMR indicates poor survival in patients with gliomas from CGGA database. **A** Kaplan–Meier survival curve analysis of overall survival(OS) showed that high HMMR expression correlated to poor prognosis of gliomas patients from CGGA database. **B-D** The association of HMMR expression with WHO grade (**B**), IDH status (**C**), and 1p/19q codeletion (**D**) in gliomas from CGGA database. **E** A nomogram that integrates HMMR and other prognostic factors in gliomas from CGGA database. **F** The calibration curve of the nomogram.*p < 0.05, **p < 0.01, ***p < 0.001

To further validate the prognostic value of HMMR mRNA expression in gliomas, we included the mRNAseq_693 dataset from the CGGA database for analysis. A total of 693 patients were included in the analysis and detailed clinicopathological features were shown in Table 1. Survival analysis showed that the high HMMR was associated with worse OS (HR = 2.21(1.80–2.71), log-rank *P* < 0.001) (Fig. 4A). High mRNA expression of HMMR was closely associated with high grade of WHO (Fig. 4B), IDH wild type (Fig. 4C) and intact 1p /19q (Fig. 4D). Univariate and multivariate COX regression analyses similarly demonstrated that HMMR was an independent prognostic factor for glioma (Table S1). Then, a nomogram of OS was created to incorporate HMMR and other prognostic variables (age, WHO grade, IDH status, 1p/19q codeletion) (Fig. 4E) with a Cindex of 0.755 (Fig. 4F).

### Functional enrichment analysis for high and low HMMR expression in gliomas patients

**Fig. 5:**
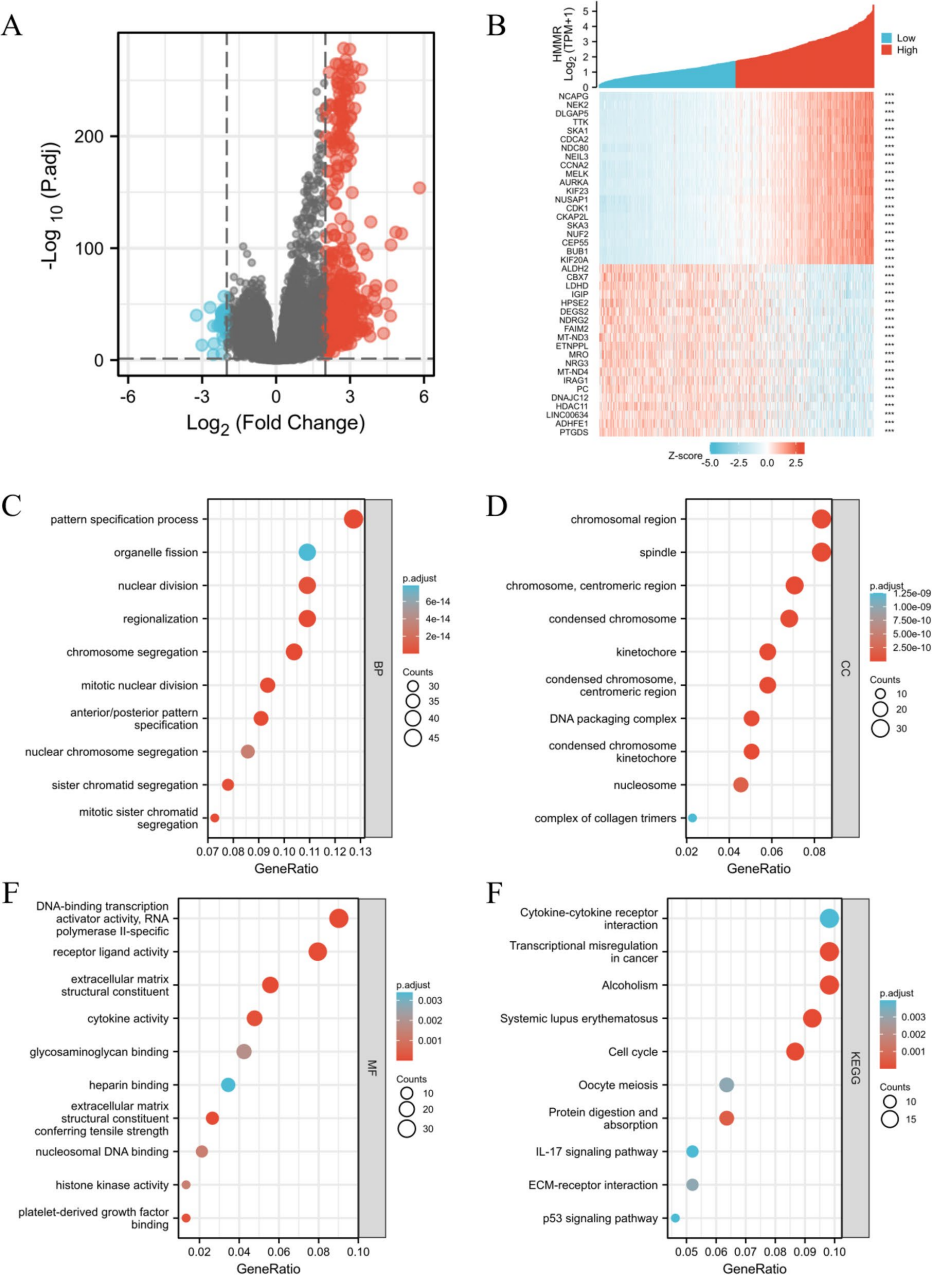
Functional enrichment analysis for HMMR expression in gliomas patients from TCGA database. **A** Volcano Plot of differentially expressed genes (DEGs) screened based on HMMR expression.** B** The top 20 positively correlated genes and the top 20 negatively correlated genes cotranscript with HMMR in gliomas. **C-E** Enrichment analysis showed the biological processes(BP) (**C**), cellular components(CC) (**D**), molecular function(MF) (**E**) and KEGG pathway analysis (**F**) of DEGs screened based on HMMR expression. *p < 0.05, **p < 0.01, ***p < 0.001

**Fig. 6:**
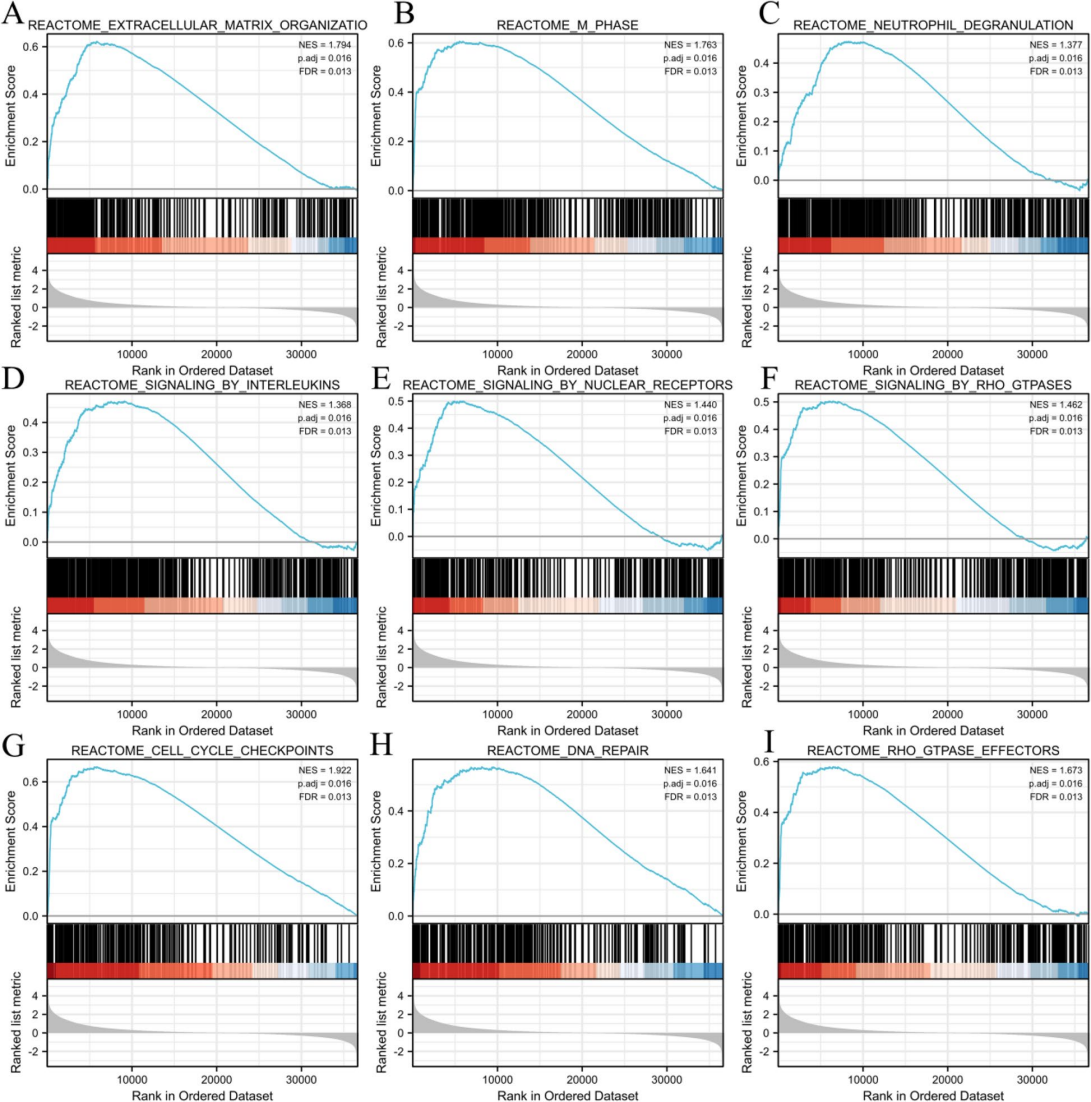
GSEA enrichment analysis results. extracellular matrix organization (**A**), m-phase (**B**), neutrophil degranulation (**C**), signaling by interleukins (**D**), signaling by nuclear receptors (**E**), signaling by RHO GTPases (**F**), cell cycle checkpoints (**G**), DNA repair (**H**), RHO GTPase effectors (**I**). were enriched mainly in HMMR-related gliomas. NES, normalized enrichment score; FDR, false discovery rate

We analyzed the DEmRNAs in high and low HMMR expression gliomas samples to further explore the potential mechanisms of HMMR that lead to gliomas progression. The results that were shown in a Volcano Plot (Fig. 5A) and heat-map (Fig. 5B) identified a total of 412 DEmRNAs, of which 373 were highly expressed and 39 were low expressed based on HMMR expression (Further detailed information were provided in Table S2). For DEmRNAs, we conducted enrichment analysis of the biological process (BF), cellular component (CC) and molecular function (MF) and kyoto encyclopedia of genes and genomes (KEEG)(Further detailed information were provided in Table S3). Significant results of enrichment analysis are shown in Fig. 5C-F. Additionally, we identified the key pathways associated with HMMR by a GSEA analysis,which found that 266 data sets satisfified the criteria of an adjusted *P*-value < 0.05 and FDR < 0.05 (Further detailed information were provided in Table S4). The top 9 most significant enrichment pathways are extracellular matrix organization (Fig. 6A), m-phase (Fig. 6B), neutrophil degranulation (Fig. 6C), signaling by interleukins (Fig. 6D), signaling by nuclear receptors (Fig. 6E), signaling by RHO GTPases (Fig. 6F), cell cycle checkpoints (Fig. 6G), DNA repair (Fig. 6H), RHO GTPase effectors (Fig. 6I).

### Correlation of HHMR expression level with immune cell infiltration in gliomas

**Fig. 7:**
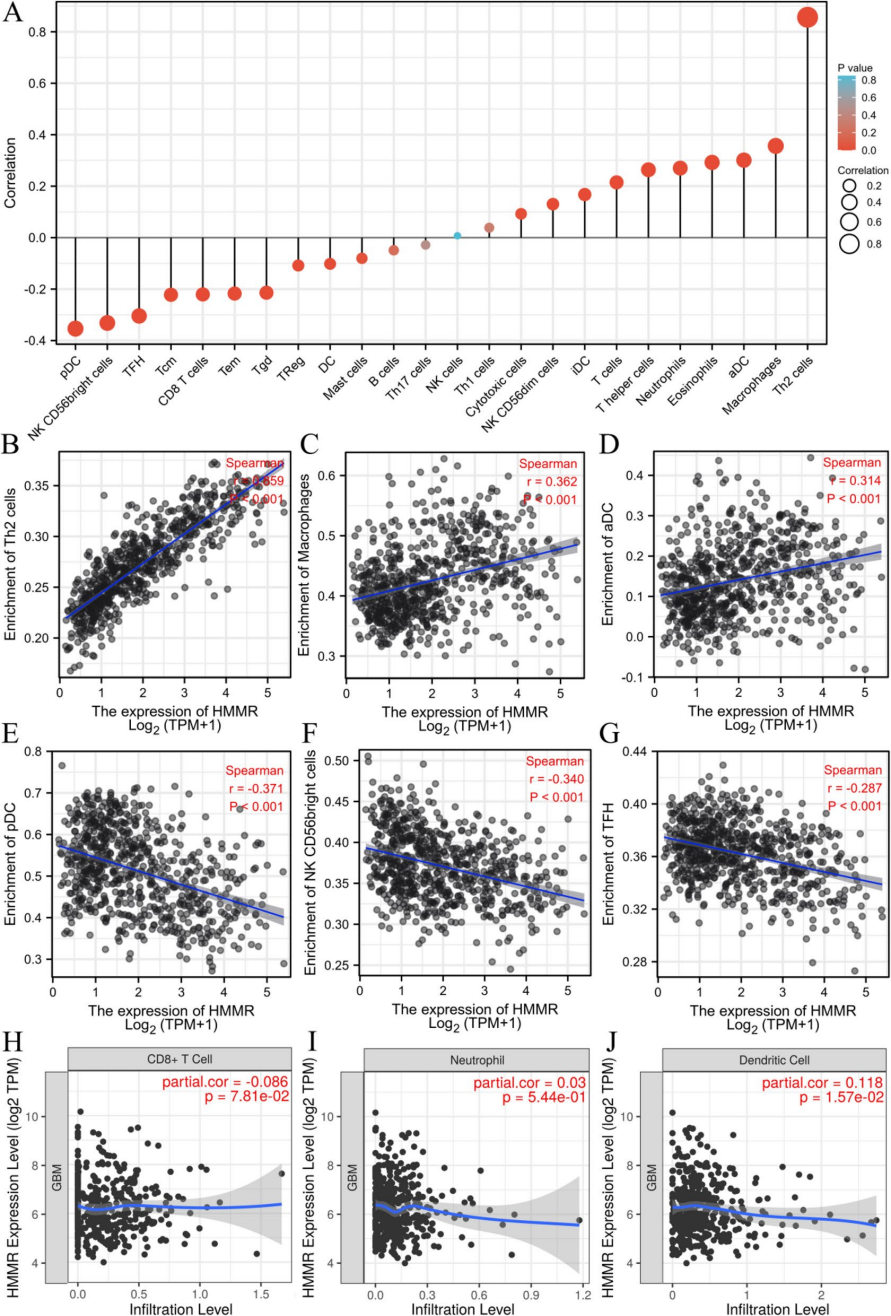
Association analysis of HMMR expression and immune infiltration in gliomas patients. **A** The association between HMMR expression and 24 tumor-infiltrating lymphocytes. **B-D** The positive correlation of HMMR expression with immune infiltration level of Th2 cells (**B**), macrophages (**C**), and aDC (**D**). **E–G** The negative correlation of HMMR expression with immune infiltration level of pDC cells (**E**), NK CD56 bright cells (**F**), and TFH (**G**). **H-J **Dendritic cell (**J**) was positively correlated with HMMR expression and CD8 + T cell (**H**), Neutrophil (**I**) had no significant correlationship with HMMR expression through TIMER software analysis

One study suggested that HMMR may participate in tumor immune response while promoting the progression of head and neck squamous cell carcinoma. To further study the relationship between HHMER and gliomas progression, we analyzed the relationship between HMMR mRNA expression and immune cells infiltration level in glioma samples by ssGSEA. The correlation between immune cell infiltration and HMMR mRNA expression was shown in Fig. 7A. Th2 cells (*R* = 0.856, *p* < 0.01, Fig. 7B), Macrophages (*R* = 0.356, *p* < 0.01, Fig. 7C) and aDC (*R* = 0.301, *p* < 0.01, Fig. 7D) were significantly positively correlated with HMMR mRNA expression, while pDC (*R* = -0.354, *p* < 0.01, Fig. 7E), NK CD56 bright cells (*R* = -0.332, *p* < 0.01, Fig. 7F) and TFH (*R* = -0.304, *p* < 0.01, Fig. 7G) were significantly negatively correlated. To verify the above results, we found that Dendritic cell (*R* = 0.118, *p* = 0.0157, Fig. 7J) were positively correlated with HMMR mRNA expression and Neutrophil (*R* = 0.03, *p* = 0.544, Fig. 7I), CD8 + T cell (*R* = -0.086, *p* = 0.0781, Fig. 7H) had no significant correlationship with HMMR mRNA expression through TIMER software analysis.

### Construction of the lncRNA-miRNA-mRNA triple regulatory network

**Fig. 8:**
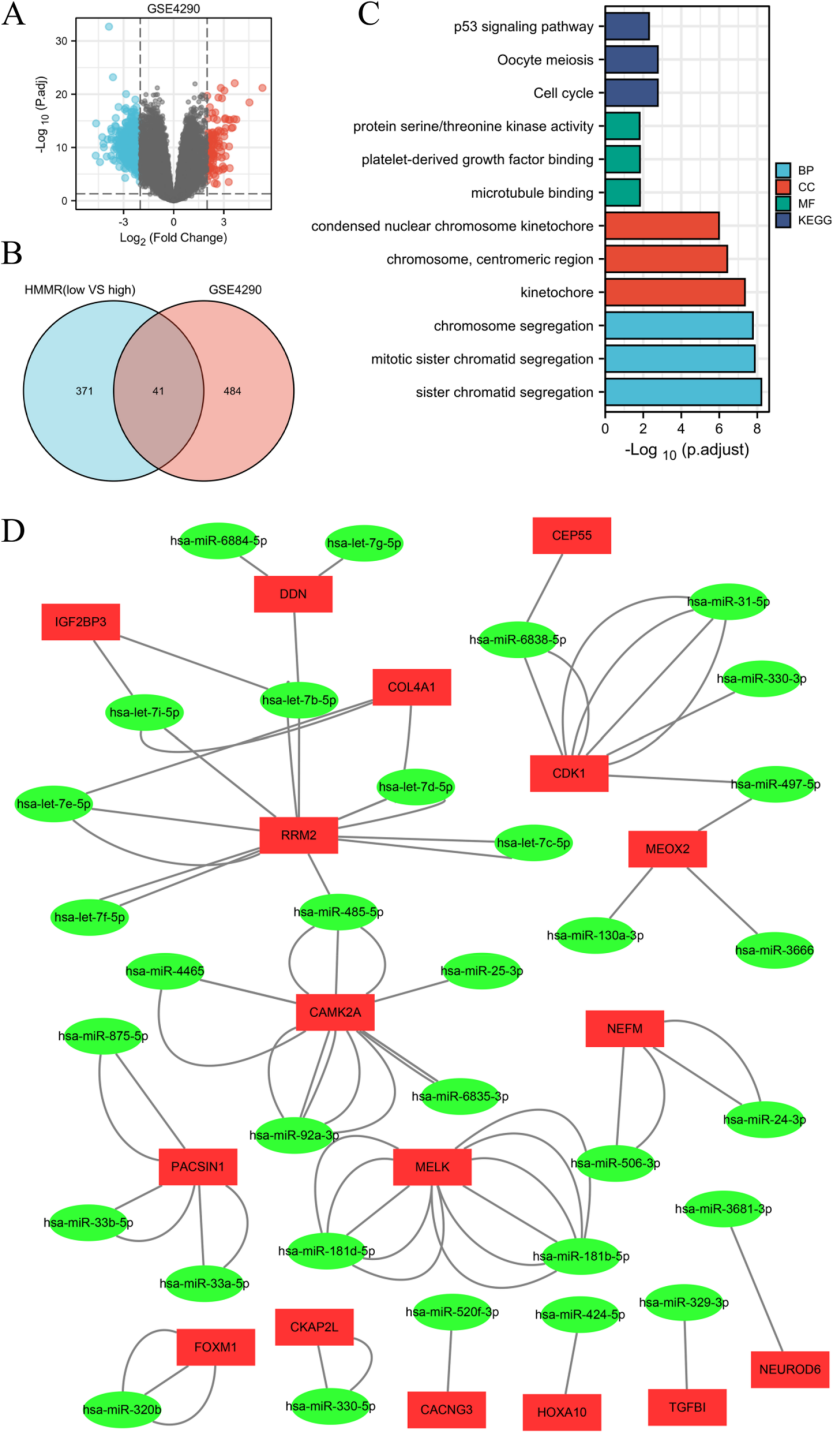
Identification of differentially expressed genes (DEGs) in GSE4290 and potential DEGs-miRNA network. **A** Volcano Plot of differentially expressed genes (DEGs) screened between gliomas and control samples from GSE4290. **B** Venn diagram of DEGs based on HMMR expression and GSE4290. **C** Enrichment analysis of overlapped DEGs. **D** Interaction network between overlapped DEGs and its targeted miRNAs. The ellipses denote miRNAs, rectangles denote mRNAs

The lncRNA-miRNA-mRNA triple regulatory network connected to HMMR could be employed as a potential prognostic model for glioma patients, according to the above findings. We did a joint study in the high and low HMMR expression groups, as well as in gliomas and control groups from GSE4290, to establish the network in gliomas. First, according to the *p* < 0.05 and |log2FC|> 2, 525 DEmRNAs were screened from GSE4290 dataset including 105 were upregulated and 420 were downregulated (Fig. 8A) (Further detailed information were provided in Table S5). A total 41 DEmRNAs were overlapped between high and low HMMR expression groups and GSE4290 (Fig. 8B) (Further detailed information were provided in Table S6). Enrichment analysis of 41 DEmRNAs were shown in Fig. 8C (Further detailed information were provided in Table S7). DEmRNAs-miRNA analysis was performed by using miRWalk 2.0 software. The crosslinked miRNAs were selected by miRWalk and TargetScan databases to ensure the accuracy and reliability of the results. Seventeen DEmRNAs and 32 potential miRNA were mined and constructed network (Fig. 8D)(Further detailed information were provided in Table S8).

**Fig. 9:**
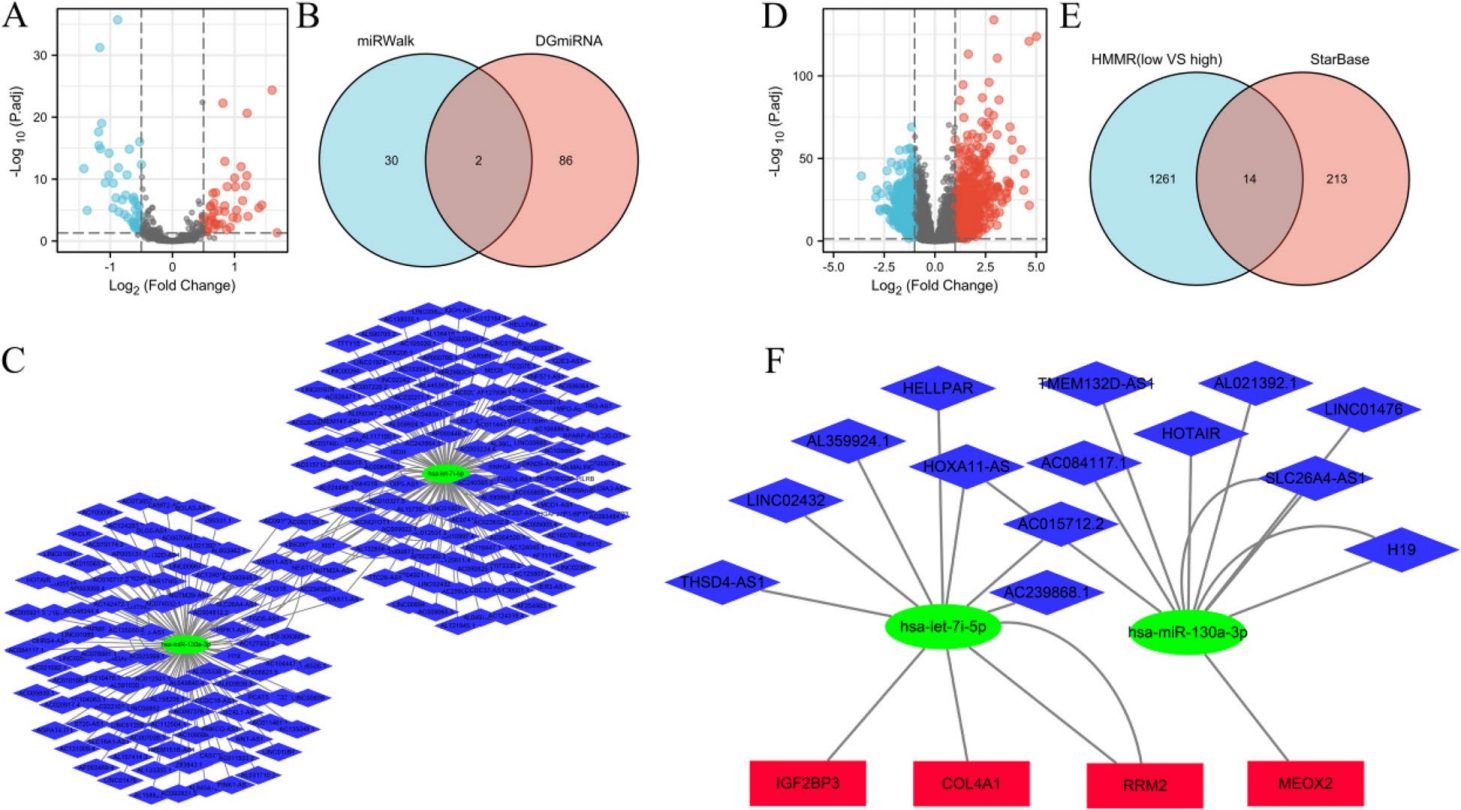
Triple regulatory network of overlapped differentially expresseddifferentially expressed lncRNAs (DELs), differentially expresseddifferentially expressed miRNAs (DEMs), and differentially expresseddifferentially expressed genes (DEGs). **A** Volcano Plot of DEMs screened based on HMMR expression. **B** Venn diagram of DEMs based on HMMR expression and overlapped DEGs’ targeted miRNAs. (**C**) Interaction network between overlapped DEMs and its targeted lncRNAs. **D** Volcano Plot of DELs screened based on HMMR expression. **E** Venn diagram of DELs based on HMMR expression and overlapped DEMs’ targeted lncRNAs. **F** Triple regulatory network of overlapped DELs, DEMs, and DEGs. The ellipses denote miRNAs, rectangles denote mRNAs, and diamonds denote lncRNAs

Then, according to the *p* < 0.05 and |log2FC|> 0.5, 88 DEmiRNAs were screened based on HMMR expression including 43 were upregulated and 45 were downregulated (Fig. 9A) (Further detailed information were provided in Table S9). Two miRNAs (hsa-let-7i-5p and hsa-miR-130a-3p) were overlapped between 88 DEmiRNAs and 32 potential miRNAs (Fig. 9B). Potential 227 lncRNAs interacted with 2 overlapped DEmiRNAs were screened in Starbase and constructed network (Fig. 9C).

In addition, according to the *p* < 0.05 and |log2FC|> 1, 1275 DElncRNAs were screened based on HMMR expression including 769 were upregulated and 506 were downregulated (Fig. 9D) (Further detailed information were provided in Table S10). Fourteen lncRNAs were overlapped between 1275 DEmiRNAs and 227 potential lncRNAs (Fig. 9E). Finally, 14 DElncRNAs, 2 DEmiRNAs, and 4 DEmRNAs were screened to construct triple regulatory network (Fig. 9F).

### Construction of ceRNA network

**Fig. 10:**
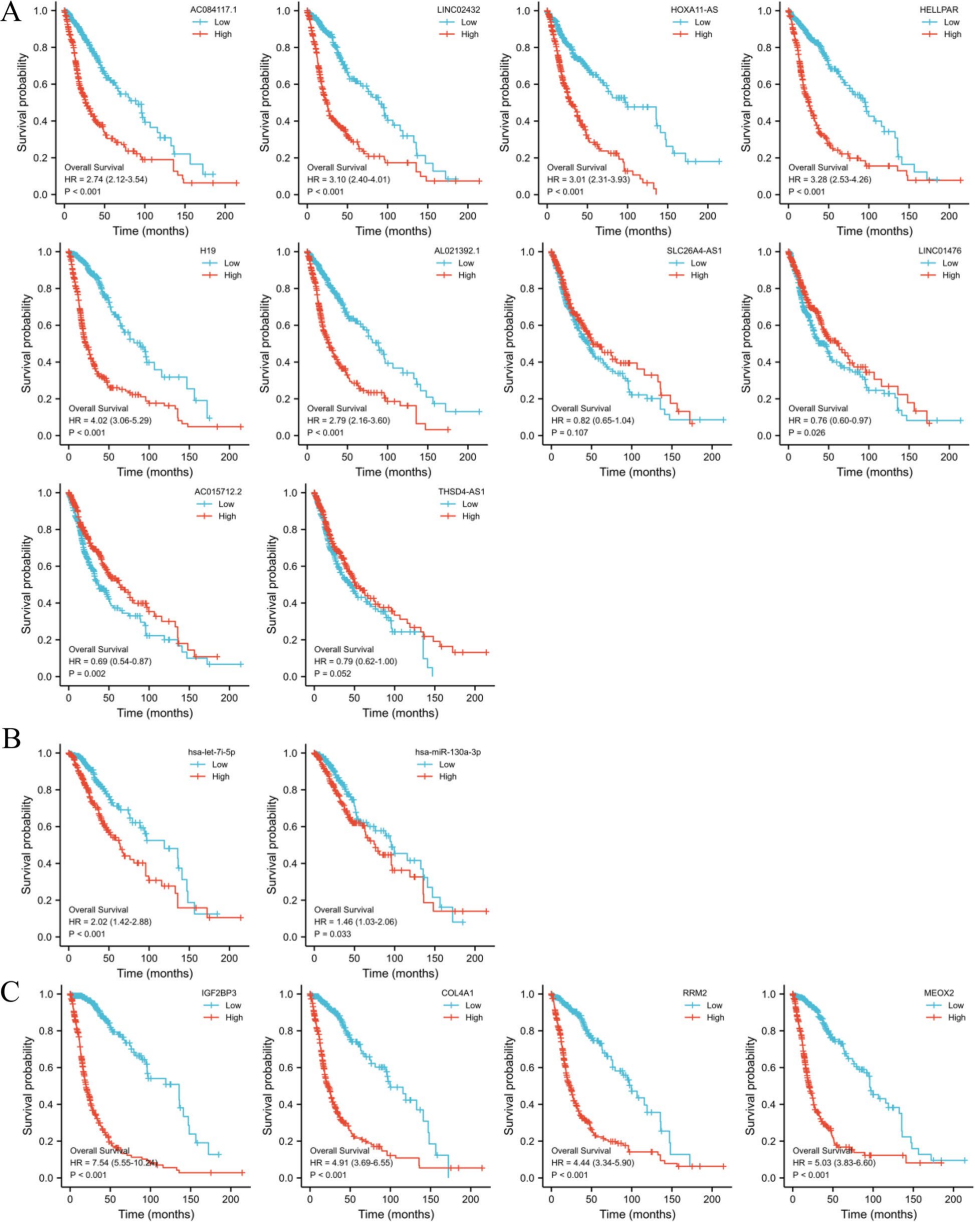
Overall survival analysis for the lncRNAs (**A**), miRNAs (**B**), and mRNA (**C**) in the triple regulatory network

We performed OS study of gliomas patients using Kaplan–Meier analysis and a log-rank test to see if these RNAs were connected with prognosis. Survival analysis was performed on 10 lncRNAs (AL359924.1, HOTAIR, TMEM132D-AS1, and AC239868.1 could not be analyzed because the number of zeros in the sequence-data was more than half), and high levels of AC084117.1, LINC02432, HOXA11-AS, HELLPAR, H19, and AL021392.1 were related to poor prognosis of gliomas patients (Fig. 10A). All 2 miRNA (Fig. 10B) and 4 mRNA (Fig. 10C) were related to prognosis.

**Fig. 11:**
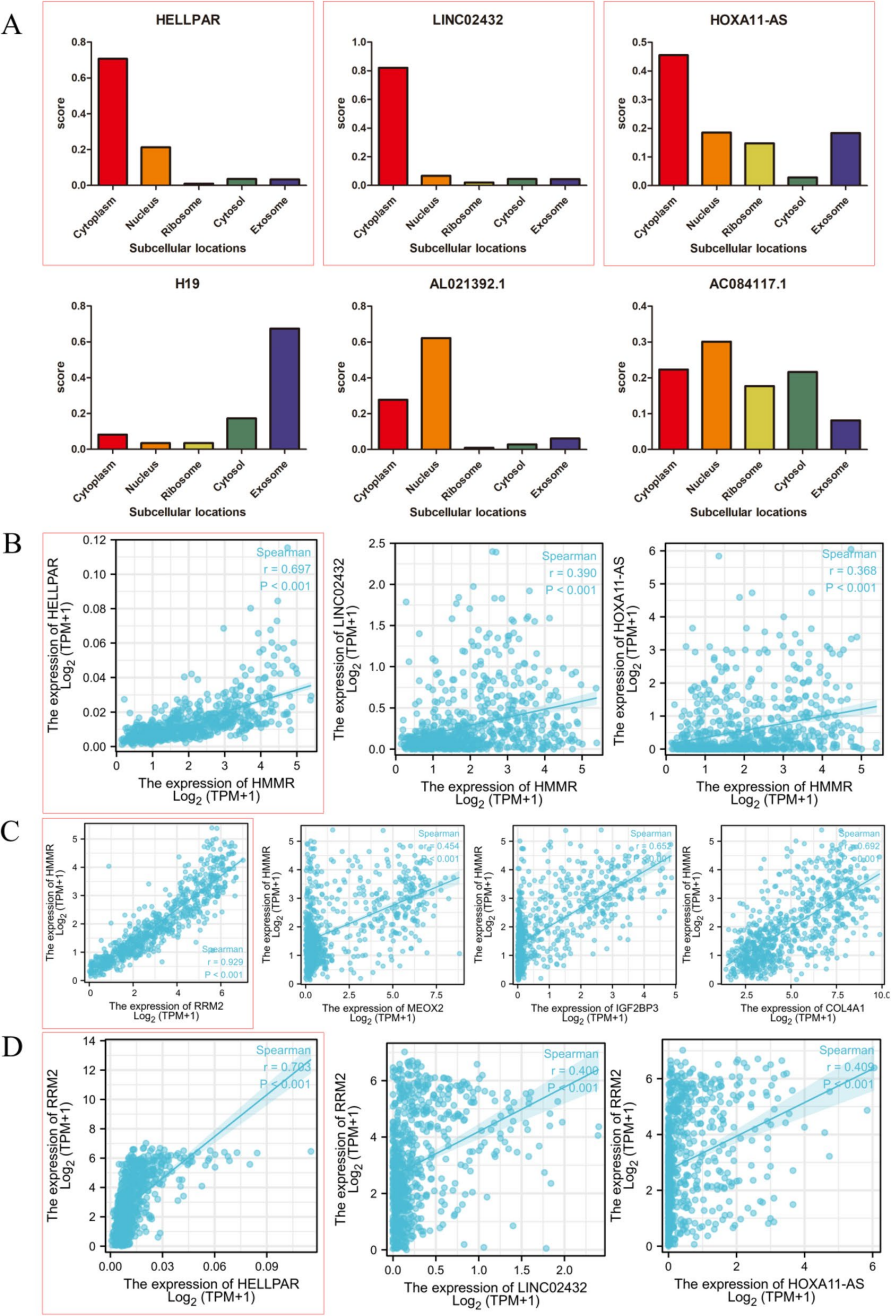
Hub lncRNA and mRNA selection. **A** The cellular localization of six lncRNAs was predicting by lncLocator, HELLPAR, LINC02432 and HOXA11-AS were mainly located in cytoplasm. **B** Correlation analysis between three lncRNAs and HMMR, the *r* between HELLPAR and HMMR was close to 0.7. **C** Correlation analysis between four mRNA and HMMR, the *r* between RRM2 and HMMR exceeded 0.9. **D** Correlation analysis between three lncRNAs and RRM2, the *r* between HELLPAR and RRM2 exceeded 0.7

Furthermore, because lncRNA cellular location determines the underlying mechanisms, we used the lncLocator to examine the subcellular localization of the 6 lncRNAs. As shown in Fig. 11A, HELLPAR, LINC02432, and HOXA11-AS were mainly located in the cytoplasm. Correlation analysis shown HELLPAR had the highest correlation coefficient with HMMR (Fig. 11B) among the three lncRNAs and RRM2 had had the highest correlation coefficient with HMMR (Fig. 11C) among the four DEmRNAs. Meanwhile, the correlation coefficient between HELLPAR and RRM2 exceeded 0.7 (Fig. 11D).

**Fig. 12:**
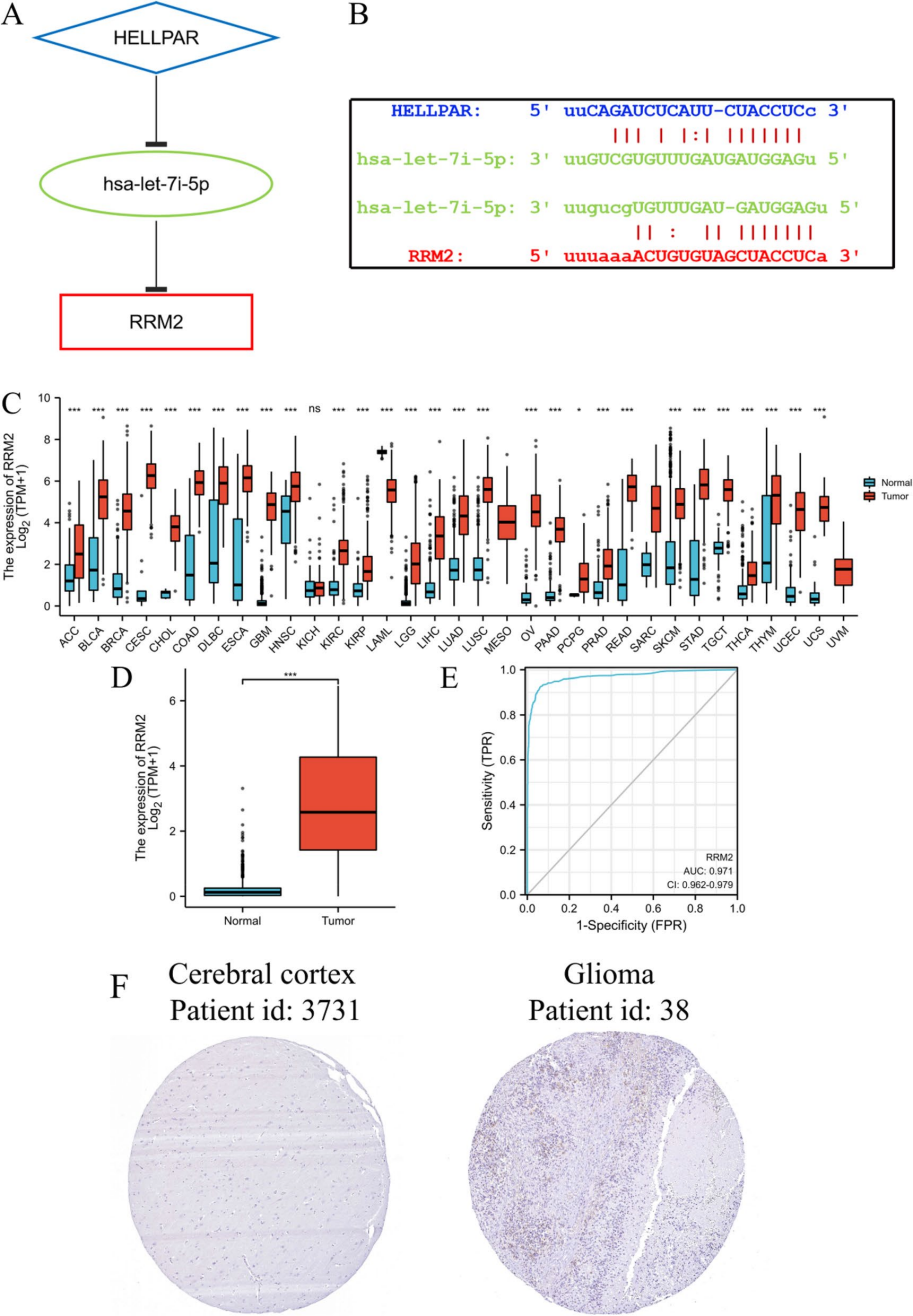
Construction of the ceRNA network and relationship between RRM2 expression and gliomas. **A** Schematic model of ceRNA (HELLPAR-hsa-let-7i-5p-RRM2). **B** Base pairing between hsa-let-7i-5p, HELLPAR and RRM2 were predicted by StarBase. **C** RRM2 expression levels in different tumor types from TCGA and GTEx database. **D** RRM2 mRNA expression was significantly upregulated in gliomas tissues compared to normal tissues. **E** Diagnostic value of RRM2 expression in gliomas. **F** RRM2 protein expression in gliomas tissues determined using HPA. ***p < 0.001, ns, no statistical difference

These findings suggest that HELLPAR acts as a ceRNA to increase RRM2 expression by sponging hsa-let-7i-5p. Thus, a HELLPAR-hsa-let-7i-5p-5p-RRM2 ceRNA network was constructed (Fig. 12A). StarBase anticipated that the target sites in the HELLPAR and RRM2 would pair with hsa-let-7i-5p (Fig. 12B).

### The prognostic significance of RRM2 in gliomas patients

**Fig. 13:**
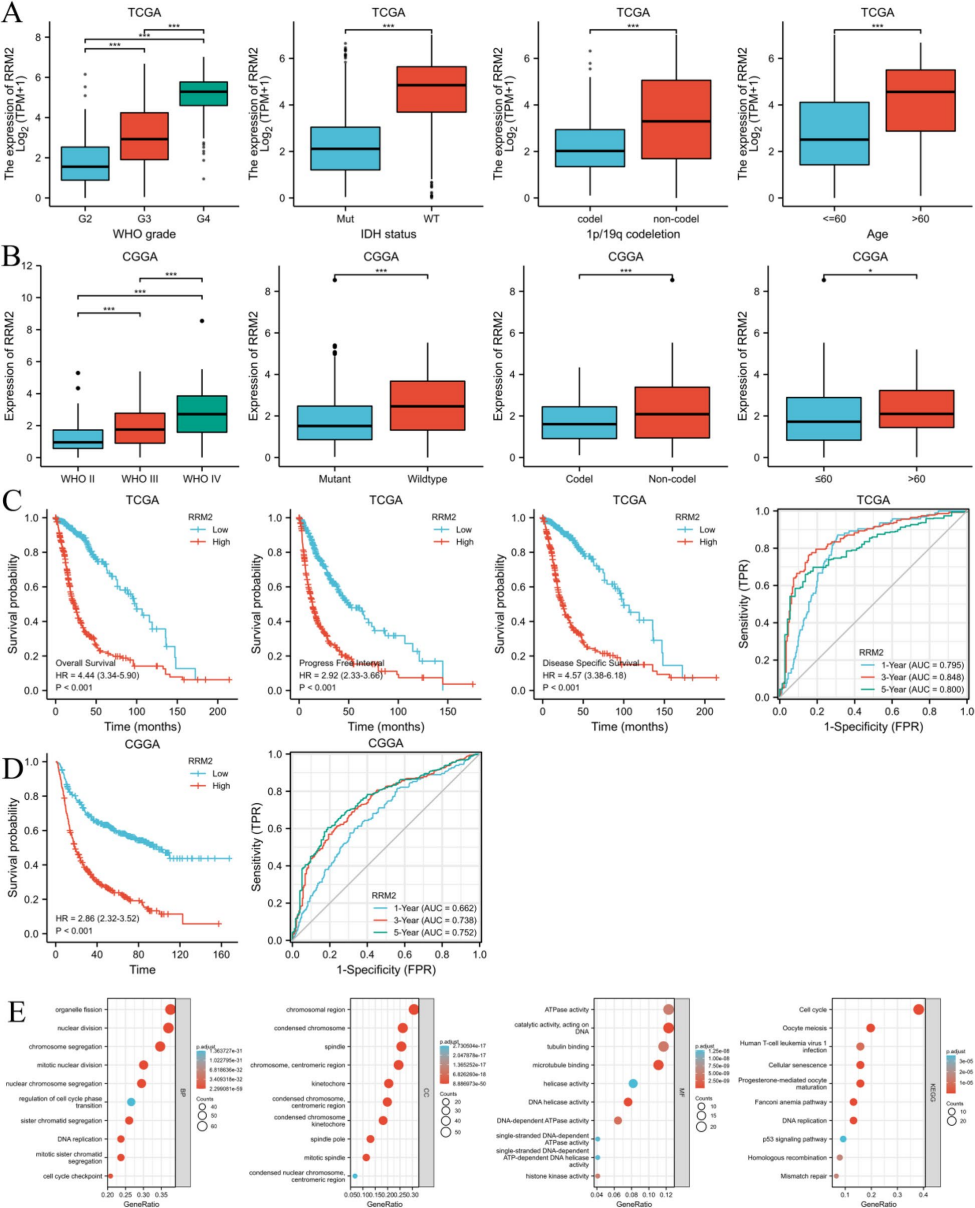
The association of RRM2 expression with WHO grade, IDH statu, 1p/19q codeletion, and age in gliomas from TCGA database (**A**) and CGGA database (**B**). Kaplan–Meier survival curve analysis and time-dependent ROC analysis showed that high RRM2 expression correlated to poor prognosis of gliomas patients from TCGA database (**C**) and CGGA database (**D**). Enrichment analysis of the correlated genes of RRM2 in gliomas including biological processes (BP), cellular components (CC), molecular function (MF) and KEGG pathway analysis (**E**)

RRM2 exhibited distinct expression profiles in various tumors and was significantly upregulated in gliomas (Fig. 12C,D). The effectiveness of RRM2 in differentiating gliomas from normal tissues was assessed by ROC analysis, with an estimated AUC of 0.971 (95% CI: 0.962–0.979; Fig. 12E). The immunohistochemical analysis of RRM2 was positive in gliomas, but negative in normal tissues (Fig. 12F). High RRM2 mRNA expression had a significant correlation with higher WHO grade, IDH wild type, non-codel of 1p19q, and advanced age (Fig. 13A, B). Compared with RRM2 low expression group, RRM2 high expression group had worse OS, DSS, and PFI from TCGA database (Fig. 13C), and worse OS from CGGA database (Fig. 13D). Time-dependent ROC analysis of RRM2 expression in gliomas patients suggusted that RRM2 could well predict the prognosis of gliomas patients at 1, 3 and 5 years (Fig. 13C, D). Furthermore, multivariate COX hazard regression analysis demonstrated that high RRM2 expression was an independent predictive factor for OS (Table S11).

Enrichment analysis of the correlated genes (correlation coefficient over 0.8) of RRM2 in gliomas were also performed (Fig. 13E) (Further detailed information were provided in Table S12 and S13).

### The prognostic significance of HELLPAR in gliomas patients

High HELLPAR expression level had a significant correlation with higher WHO grade, IDH wild type, non-codel of 1p19q, and advanced age (Fig. S1A). Compared with low expression group, HELLPAR high expression group had worse OS, DSS, and PFI from TCGA database (Fig. S1B). A time-dependent ROC analysis of HELLPAR expression in glioma patients suggested that HELLPAR may accurately predict glioma patients' prognosis at 1, 3, and 5 years (Fig. S1B). Furthermore, multivariate cox hazard regression analysis demonstrated that elevated HELLPAR expression was an independent predictive predictor for OS (Table S14).

## Discussion

Gliomas is the most common malignancy of the CNS, and more than half of gliomas are detected at an advanced stage, accompanied by rapid malignant progression, resulting in poor prognosis and high mortality [49, 50]. Early diagnosis and effective treatment of gliomas patients can improve their prognosis and survival. As a result, early detection and identification of glioma growth, as well as the discovery of new treatment targets and the development of new therapeutic techniques, are critical [51, 52]. The rapid development of sequencing and omics technologies in recent years has given researchers greater opportunity to learn more about the pathophysiology of gliomas and to investigate diagnostic and therapeutic targets [53–55]. Previous research has linked high HMMR expression to a bad prognosis in a variety of malignancies, such as breast, colorectal, gastric, endometrial, prostate, and multiple myeloma [56–61]. Furthermore, these investigations imply that HMMR is linked to tumor growth and metastasis. Several human malignancies, including lung cancer, breast cancer, and hepatocellular carcinoma, have been revealed to contain ceRNA regulatory networks implicated in their genesis and progression [62–64]. However, the prognostic usefulness of HMMR in gliomas has not been thoroughly examined, and just a few research have focused on the integrated ceRNA regulation network as a means of predicting glioma prognosis.

In this study, first, we comprehensively analyzed the expression of HMMR in pan-cancer using bioinformatics and multiple databases, and found that HMMR is abnormally expressed in most cancers including gliomas, which is consistent with current literature reports [14, 65, 66]. The expression of HMMR was significantly increased in gliomas and was significantly associated with high-grade gliomas, advanced age, wild-type IDH and non-codeletion of 1p19q suggesting that HMMR might associated with gliomas disease progression. Meanwhile, HMMR was a useful glioma diagnostic marker, and its AUC was 0.937. HMMR was found to be an independent risk factor for glioma prognosis in both univariate and multivariate Cox regression analyses. Because HMMR expression was a powerful predictive predictor, we created a nomogram that combined HMMR expression and clinical data, and the nomogram predicted 1-, 3-, and 5-year OS in glioma patients more correctly. In addition, an increasing number of studies have discovered that immune cells play a significant function in the tumor microenvironment and play a role in tumor formation and development [67–69]. By performing immune infiltration analysis by the ssGSEA method, we discovered a link between HMMR expression and immune infiltration in gliomas, which supports the findings of the current study [70–73]. We investigated DEmRNAs in high and low HMMR expressing gliomas samples and performed functional enrichment analysis to fully understand the function of HMMR in gliomas. It was found that HMMR may be involved in glioma through cell cycle pathways, nuclear division, and other pathway. The result of GSEA revealed that HMMR may be related to the extracellular matrix organization pathway, m-phase pathway, and cell cycle checkpoints pathway. HMMR has also been implicated in a variety of biological functions, including cell proliferation, cycle control, migration, and invasion, according to previous research [74–77]. To characterize the level of immune infiltration in gliomas, we assessed the association between HMMR and immune cell populations based on transcriptomic data. The results showed that the expression of HMMR was closely related to immune infiltration, with the most positive correlation with Th2 cells, macrophages, and aDC, and the most negative correlation with pDC cells, NK CD56bright cells, and TFH. These findings suggested that HMMR might play an important role in regulating immune functions in gliomas.

Considering the important role of HMMR in predicting gliomas prognosis and the potentially important role of ceRNA network in gliomas disease progression. Then, we constructed a HMMR-related ceRNA network. Combined analysis of DEmRNA from HMMR expression group and GSE4290 resulted in 41 overlapped of DEmRNAs. The enrichment analysis of these 41 DEmRNAs found that the cell cycle and mitotic pathways were enriched which consisted with previous results. Potential interaction miRNAs and lncRNAs were predicted by miRWalk and StarBase, respectively. After analyzed with DEmiRNAs and DElncRNAs, a total 4 mRNAs, 2 miRNAs, and 14 lncRNAs were selected to construct triple regulatory network. This triple regulatory network was also subjected to a survival analysis. We also performed subcellular localization analysis of the lncRNAs in the network because the interactions in the ceRNA network exist solely in the cytoplasm. Finally, combined the results of correlation analysis, the HELLPAR-hsa-let-7i-5p-RRM2 overexpressed ceRNA network was obtained. Further analysis indicated RRM2 expression was shown to be considerably higher in giomas and high RRM2 and HELLPAR expression were linked to a poor outcome in survival analysis..

Ribonucleotide reductase (RNR) is a key enzyme in DNA synthesis and has two subunits, ribonucleotide reductase subunits M1 and 2 (RRM1, RRM2) [78]. In proliferating cells, the RRM1-RRM2 holoenzyme supplies deoxyribonucleoside triphosphates (dNTPs) for nuclear DNA replication and repair in S-phase, as well as dNTPs for mitochondrial DNA replication and repair [79, 80]. RRM2 is commonly expressed in cancer, and it is thought to be an oncogene and a potential cancer treatment target [81, 82]. Gandhi et al*.* found the YBX1-RRM2-TYMS-TK1 axis, which governed nucleotide metabolism, could be modulated by lincNMR to control tumor cell growth [83]. Xie et al*.* reported miR-520a could inhibit lung cancer progression through suppressing RRM2 expression and Wnt signaling pathway activation [84]. In glioma disease, RRM2 can promote human glioblastoma cell proliferation, migration and invasion and RRM2 expression controlled by BRCA1 can protect glioblastoma cells from endogenous replication stress while also increasing tumorigenicity [85, 86]. At the same time, through the functional enrichment analysis of RRM2-related genes, we found that cell cycle, nuclear division, and mitosis pathway were significantly enriched, suggesting that RRM2 may be involved in glioma tumorigenesis and progression by regulating cell proliferation, which is consistent with the current RRM2 function. Given that this result was consistent with previous enrichment analyses, we speculated that the HMMR and RRM2 were involved in glioma disease progression by regulating cell cycle and proliferation.

MicroRNAs can control gene expression by recognizing homologous sequences and interfering with transcription, translation, and epigenetic processes. The regulatory role of miRNAs on tumors has been confirmed by many studies [87, 88]. Yang et al*.* found the HDAC6-hsa-let-7i-5p-TSP1 pathway can regulate neoplastic and antiphagocytic behaviors of hepatocellular carcinoma [89]. Liu et al*.* performed bioinformatics analysis and concluded that hsa-let-7i-5p functions as a tumor promoter in clear cell renal cell carcinoma and facilitates cell proliferation, migration and invasion by targeting HABP4 [90]. And GALE can be regulated by hsa-let-7i-5p to inhibit human glioblastoma growth [91]. However, whether hsa-let-7i-5p participates in the development of gliomas by regulating RRM2 still needs further research to confirm.

Although studies have shown that HELLPAR affects the transition of the extravillous trophoblast (EVT) from a proliferative to an invasive state, the role of HELLPAR in glioma has not been reported and further experiments are needed to verify its specific function [92, 93].

This study also has several limitations. First, most of the data used for the analysis were mined from public databases and validated in in vitro experiments; however, some results may require further validation in future studies. Second, limited by the difficulty of obtaining normal brain tissue, the GSE4290 dataset analyzed the brain tissue of epilepsy patients as a control group, and the immunohistochemical images of normal cerebral cortex tissue were obtained from HPA, which may have an impact on the results. Third, the binding affinity of lncRNAs, miRNAs, and mRNAs retrieved from the database should be studied further in the lab. Finally, we need to further study the function and mechanism of HMMR and theHELLPAR/ RRM2 axis in gliomas by experiments.

## Conclusion

In conclusion, this study provides multi-layered and multifaceted evidence for the importance of HMMR in gliomas and establishes a HMMR related ceRNA (HEELPAR-hsa-let-7i-5p-RRM2) overexpressed network of gliomas, which is better for understanding the link among lncRNA-miRNA-mRNA. These findings suggest potential targets for gliomas treatment.

## Supplementary Information

**Additional file 1: Table S1**. Univariate and multivariate analysis of demographic and clinical characteristics that correlate with OS of gliomas patients (CGGA database). **Table S2.** DEmRNAs in high and low HMMR expression gliomas samples (TCGA database). **Table S3.** Functional enrichment analysis for DEmRNAs based on HMMR expression. **Table S4.** GSEA analysis associated with HMMR. **Table S5.** DEmRNAs in GSE4290. **Table S6.** overlapped DEmRNAs. **Table S7.** Enrichment analysis of 41 DEmRNAs. **Table S8.** DEmRNAs-miRNA analysis. **Table S9.** DEmiRNAs in high and low HMMR expression gliomas samples (TCGA database). **Table S10.** DElncRNAs in high and low HMMR expression gliomas samples (TCGA database). **Table S11.** Univariate and multivariate analysis of demographic and clinical characteristics that correlate with OS of gliomas patients (TGGA database). **Table S12.** Correlation analysis of RRM2 (TCGA database). **Table S13.** Enrichment analysis of the correlated genes (correlation coefficient over 0.8) of RRM2 in gliomas. **Table S14.** Univariate and multivariate analysis of demographic and clinical characteristics that correlate with OS of gliomas patients (TGGA database).

**Additional file 2: Figure S1.** The association of RRM2 expression with WHO grade, IDH statu, 1p/19q codeletion, and age in gliomas from TCGA database (A). Kaplan-Meier survival curve analysis and time-dependent ROC analysis showed that high RRM2 expression correlated to poor prognosis of gliomas patients from TCGA database (B). **Figure S2.** Correlation analysis of the ceRNA network.

## Data Availability

Datasets of this study are available in the GEO database (https://www.ncbi.nlm.nih.gov/geo/), UCSCXENA database (https://xenabrowser.net/datapages/), the Human Protein Atlas database (https://www.proteinatlas.org/), and CGGA database (http://www.cgga.org.cn/).

## References

[1] Tykocki T, Eltayeb M (2018). Ten-year survival in glioblastoma. A systematic review J Clin Neurosci.

[2] Liu CA, Chang CY, Hsueh KW, Su HL, Chiou TW, Lin SZ, et al. Migration/invasion of malignant gliomas and implications for therapeutic treatment. Int J Mol Sci. 2018;19. 10.3390/ijms19041115 10.3390/ijms19041115 PMC5979613 29642503

[3] Desland FA, Hormigo A. The cns and the brain tumor microenvironment: implications for glioblastoma immunotherapy. Int J Mol Sci. 2020;21. 10.3390/ijms21197358 10.3390/ijms21197358 PMC7582539 33027976

[4] Davis ME (2016). Glioblastoma: overview of disease and treatment. Clin J Oncol Nurs.

[5] Lee E, Yong RL, Paddison P, Zhu J (2018). Comparison of glioblastoma (gbm) molecular classification methods. Semin Cancer Biol.

[6] Batash R, Asna N, Schaffer P, Francis N, Schaffer M (2017). Glioblastoma multiforme, diagnosis and treatment. Recent literature review Curr Med Chem.

[7] Richardson TE, Patel S, Serrano J, Sathe AA, Daoud EV, Oliver D (2019). Genome-wide analysis of glioblastoma patients with unexpectedly long survival. J Neuropathol Exp Neurol.

[8] Johnson A, Severson E, Gay L, Vergilio JA, Elvin J, Suh J (2017). Comprehensive genomic profiling of 282 pediatric low- and high-grade gliomas reveals genomic drivers, tumor mutational burden, and hypermutation signatures. Oncologist.

[9] Quinones A, Le A (2018). The multifaceted metabolism of glioblastoma. Adv Exp Med Biol.

[10] Chen R, Smith-Cohn M, Cohen AL, Colman H (2017). Glioma subclassifications and their clinical significance. Neurotherapeutics.

[11] Brito C, Azevedo A, Esteves S, Marques AR, Martins C, Costa I (2019). Clinical insights gained by refining the 2016 who classification of diffuse gliomas with: egfr amplification, tert mutations, pten deletion and mgmt methylation. BMC Cancer.

[12] Izquierdo C, Joubert B, Ducray F (2017). Anaplastic gliomas in adults: an update. Curr Opin Oncol.

[13] Hardwick C, Hoare K, Owens R, Hohn HP, Hook M, Moore D (1992). Molecular cloning of a novel hyaluronan receptor that mediates tumor cell motility. J Cell Biol.

[14] Schwertfeger KL, Cowman MK, Telmer PG, Turley EA, McCarthy JB (2015). Hyaluronan, inflammation, and breast cancer progression. Front Immunol.

[15] Kang HG, Kim WJ, Kang HG, Chun KH, Kim SJ (2020). Galectin-3 interacts with c/ebpbeta and upregulates hyaluronan-mediated motility receptor expression in gastric cancer. Mol Cancer Res.

[16] Yang D, Ma Y, Zhao P, Ma J, He C (2021). Hmmr is a downstream target of foxm1 in enhancing proliferation and partial epithelial-to-mesenchymal transition of bladder cancer cells. Exp Cell Res..

[17] Sun Y, Li Z, Song K (2021). Ar-mtor-srf axis regulates hmmr expression in human prostate cancer cells. Biomol Ther (Seoul).

[18] Mohan P, Castellsague J, Jiang J, Allen K, Chen H, Nemirovsky O (2013). Genomic imbalance of hmmr/rhamm regulates the sensitivity and response of malignant peripheral nerve sheath tumour cells to aurora kinase inhibition. Oncotarget.

[19] Yang J, Du X (2013). Genomic and molecular aberrations in malignant peripheral nerve sheath tumor and their roles in personalized target therapy. Surg Oncol.

[20] Tilghman J, Wu H, Sang Y, Shi X, Guerrero-Cazares H, Quinones-Hinojosa A (2014). Hmmr maintains the stemness and tumorigenicity of glioblastoma stem-like cells. Cancer Res.

[21] Wienholds E, Plasterk RH (2005). Microrna function in animal development. Febs Lett.

[22] Wilusz JE (2016). Long noncoding rnas: re-writing dogmas of rna processing and stability. Biochim Biophys Acta.

[23] Tong Y, Yang L, Yu C, Zhu W, Zhou X, Xiong Y (2020). Tumor-secreted exosomal lncrna pou3f3 promotes cisplatin resistance in escc by inducing fibroblast differentiation into cafs. Mol Ther Oncolytics.

[24] Zhang Y, Huang YX, Wang DL, Yang B, Yan HY, Lin LH (2020). Lncrna dscam-as1 interacts with ybx1 to promote cancer progression by forming a positive feedback loop that activates foxa1 transcription network. Theranostics.

[25] Zhu J, Zhang Y, Chen X, Bian Y, Li J, Wang K (2022). The emerging roles of linc00665 in human cancers. Front Cell Dev Biol..

[26] Teng F, Zhang JX, Chang QM, Wu XB, Tang WG, Wang JF (2020). Lncrna mylk-as1 facilitates tumor progression and angiogenesis by targeting mir-424-5p/e2f7 axis and activating vegfr-2 signaling pathway in hepatocellular carcinoma. J Exp Clin Cancer Res.

[27] Zhou R, Sun H, Zheng S, Zhang J, Zeng D, Wu J (2020). A stroma-related lncrna panel for predicting recurrence and adjuvant chemotherapy benefit in patients with early-stage colon cancer. J Cell Mol Med.

[28] Sheng J, He X, Yu W, Chen Y, Long Y, Wang K (2021). P53-targeted lncrna st7-as1 acts as a tumour suppressor by interacting with ptbp1 to suppress the wnt/beta-catenin signalling pathway in glioma. Cancer Lett.

[29] Tang G, Luo L, Zhang J, Zhai D, Huang D, Yin J (2021). Lncrna linc01057 promotes mesenchymal differentiation by activating nf-kappab signaling in glioblastoma. Cancer Lett.

[30] Mu M, Niu W, Zhang X, Hu S, Niu C (2020). Lncrna bcyrn1 inhibits glioma tumorigenesis by competitively binding with mir-619-5p to regulate cuedc2 expression and the pten/akt/p21 pathway. Oncogene.

[31] Fu C, Li D, Zhang X, Liu N, Chi G, Jin X (2018). Lncrna pvt1 facilitates tumorigenesis and progression of glioma via regulation of mir-128-3p/grem1 axis and bmp signaling pathway. Neurotherapeutics.

[32] Bartel DP (2004). Micrornas: genomics, biogenesis, mechanism, and function. Cell.

[33] Sharma U, Conine CC, Shea JM, Boskovic A, Derr AG, Bing XY (2016). Biogenesis and function of trna fragments during sperm maturation and fertilization in mammals. Science.

[34] Zhang N, Hu G, Myers TG, Williamson PR (2019). Protocols for the analysis of microrna expression, biogenesis, and function in immune cells. Curr Protoc Immunol..

[35] Zhang PF, Pei X, Li KS, Jin LN, Wang F, Wu J (2019). Circular rna circfgfr1 promotes progression and anti-pd-1 resistance by sponging mir-381-3p in non-small cell lung cancer cells. Mol Cancer.

[36] Shi Y, Zhang DD, Liu JB, Yang XL, Xin R, Jia CY (2021). Comprehensive analysis to identify dleu2l/taok1 axis as a prognostic biomarker in hepatocellular carcinoma. Mol Ther Nucleic Acids.

[37] Salmena L, Poliseno L, Tay Y, Kats L, Pandolfi PP. A cerna hypothesis: the rosetta stone of a hidden rna language? Cell. 2011;146:353-8. 10.1016/j.cell.2011.07.014 10.1016/j.cell.2011.07.014 PMC3235919 21802130

[38] Chan JJ, Tay Y. Noncoding rna:rna regulatory networks in cancer. Int J Mol Sci. 2018;19. 10.3390/ijms19051310 10.3390/ijms19051310 PMC5983611 29702599

[39] Mu Y, Tang Q, Feng H, Zhu L, Wang Y (2020). Lncrna ktn1as1 promotes glioma cell proliferation and invasion by negatively regulating mir5053p. Oncol Rep.

[40] Li Y, Wang X, Zhao Z, Shang J, Li G, Zhang R. Lncrna neat1 promotes glioma cancer progression via regulation of mir-98-5p/bzw1. Biosci Rep. 10.1042/BSR20200767 10.1042/BSR20200767 PMC8314435 33393590

[41] Xin J, Zhao YH, Zhang XY, Tian LQ (2020). Lncrna nfia-as2 promotes glioma progression through modulating the mir-655-3p/zfx axis. Hum Cell.

[42] Love MI, Huber W, Anders S (2014). Moderated estimation of fold change and dispersion for rna-seq data with deseq2. Genome Biol.

[43] Ritchie ME, Phipson B, Wu D, Hu Y, Law CW, Shi W (2015). Limma powers differential expression analyses for rna-sequencing and microarray studies. Nucleic Acids Res..

[44] Yu G, Wang LG, Han Y, He QY (2012). Clusterprofiler: an r package for comparing biological themes among gene clusters. OMICS.

[45] Kanehisa M, Furumichi M, Sato Y, Ishiguro-Watanabe M, Tanabe M (2021). Kegg: integrating viruses and cellular organisms. Nucleic Acids Res.

[46] Kanehisa M (2019). Toward understanding the origin and evolution of cellular organisms. Protein Sci.

[47] Hanzelmann S, Castelo R, Guinney J (2013). Gsva: gene set variation analysis for microarray and rna-seq data. BMC Bioinformatics.

[48] Li JH, Liu S, Zhou H, Qu LH, Yang JH (2014). Starbase v2.0: decoding mirna-cerna, mirna-ncrna and protein-rna interaction networks from large-scale clip-seq data. Nucleic Acids Res..

[49] Guo X, Wang T, Huang G, Li R, Da CC, Li H (2021). Rediscovering potential molecular targets for glioma therapy through the analysis of the cell of origin, microenvironment and metabolism. Curr Cancer Drug Targets.

[50] Muller BJ, Kulasinghe A, Chua B, Day BW, Punyadeera C (2020). Circulating biomarkers in patients with glioblastoma. Br J Cancer.

[51] Ross JL, Velazquez VJ, Plant A, MacDonald TJ, Becher OJ, Hambardzumyan D (2021). Tumour immune landscape of paediatric high-grade gliomas. Brain.

[52] Whitfield BT, Huse JT. Classification of adult-type diffuse gliomas: impact of the world health organization 2021 update. Brain Pathol. 2022:e13062. 10.1111/bpa.13062 10.1111/bpa.13062 PMC9245936 35289001

[53] Pienkowski T, Kowalczyk T, Garcia-Romero N, Ayuso-Sacido A, Ciborowski M. Proteomics and metabolomics approach in adult and pediatric glioma diagnostics. Biochim Biophys Acta Rev Cancer. 2022:88721. 10.1016/j.bbcan.2022.188721 10.1016/j.bbcan.2022.188721 35304294

[54] Wang QW, Zhao Z, Bao ZS, Jiang T, Zhu YJ (2021). Comprehensive analysis of multi-omics data of recurrent gliomas identifies a recurrence-related signature as a novel prognostic marker. Am J Cancer Res.

[55] Su W, Liao M, Tan H, Chen Y, Zhao R, Jin W (2021). Identification of autophagic target rab13 with small-molecule inhibitor in low-grade glioma via integrated multi-omics approaches coupled with virtual screening of traditional chinese medicine databases. Cell Prolif..

[56] Assmann V, Gillett CE, Poulsom R, Ryder K, Hart IR, Hanby AM (2001). The pattern of expression of the microtubule-binding protein rhamm/ihabp in mammary carcinoma suggests a role in the invasive behaviour of tumour cells. J Pathol.

[57] Zlobec I, Baker K, Terracciano LM, Lugli A (2008). Rhamm, p21 combined phenotype identifies microsatellite instability-high colorectal cancers with a highly adverse prognosis. Clin Cancer Res.

[58] Li H, Guo L, Li JW, Liu N, Qi R, Liu J (2000). Expression of hyaluronan receptors cd44 and rhamm in stomach cancers: relevance with tumor progression. Int J Oncol.

[59] Rein DT, Roehrig K, Schondorf T, Lazar A, Fleisch M, Niederacher D (2003). Expression of the hyaluronan receptor rhamm in endometrial carcinomas suggests a role in tumour progression and metastasis. J Cancer Res Clin Oncol.

[60] Gust KM, Hofer MD, Perner SR, Kim R, Chinnaiyan AM, Varambally S (2009). Rhamm (cd168) is overexpressed at the protein level and may constitute an immunogenic antigen in advanced prostate cancer disease. Neoplasia.

[61] Maxwell CA, Rasmussen E, Zhan F, Keats JJ, Adamia S, Strachan E (2004). Rhamm expression and isoform balance predict aggressive disease and poor survival in multiple myeloma. Blood.

[62] Ping Y, Zhou Y, Hu J, Pang L, Xu C, Xiao Y (2020). Dissecting the functional mechanisms of somatic copy-number alterations based on dysregulated cerna networks across cancers. Mol Ther Nucleic Acids.

[63] Zhao W, Geng D, Li S, Chen Z, Sun M (2018). Lncrna hotair influences cell growth, migration, invasion, and apoptosis via the mir-20a-5p/hmga2 axis in breast cancer. Cancer Med.

[64] Ni W, Zhang Y, Zhan Z, Ye F, Liang Y, Huang J (2017). A novel lncrna uc.134 represses hepatocellular carcinoma progression by inhibiting cul4a-mediated ubiquitination of lats1. J Hematol Oncol..

[65] Tang YP, Yin YX, Xie MZ, Liang XQ, Li JL, Li KZ (2021). Systematic analysis of the clinical significance of hyaluronan-mediated motility receptor in colorectal cancer. Front Mol Biosci..

[66] Mantripragada KK, Spurlock G, Kluwe L, Chuzhanova N, Ferner RE, Frayling IM (2008). High-resolution dna copy number profiling of malignant peripheral nerve sheath tumors using targeted microarray-based comparative genomic hybridization. Clin Cancer Res.

[67] Hamon P, Gerbe DTM, Classe M, Signolle N, Liu W, Bawa O, et al. Tgfbeta receptor inhibition unleashes interferon-beta production by tumor-associated macrophages and enhances radiotherapy efficacy. J Immunother Cancer. 2022;10. 10.1136/jitc-2021-003519 10.1136/jitc-2021-003519 PMC8932273 35301235

[68] Esteban-Fabro R, Willoughby CE, Pique-Gili M, Montironi C, Abril-Fornaguera J, Peix J (2022). Cabozantinib enhances anti-pd1 activity and elicits a neutrophil-based immune response in hepatocellular carcinoma. Clin Cancer Res.

[69] Braun DA, Bakouny Z, Hirsch L, Flippot R, Van Allen EM, Wu CJ (2021). Beyond conventional immune-checkpoint inhibition - novel immunotherapies for renal cell carcinoma. Nat Rev Clin Oncol.

[70] Xu Z, Chen X, Song L, Yuan F, Yan Y (2022). Matrix remodeling-associated protein 8 as a novel indicator contributing to glioma immune response by regulating ferroptosis. Front Immunol..

[71] Domingues P, Gonzalez-Tablas M, Otero A, Pascual D, Miranda D, Ruiz L (2016). Tumor infiltrating immune cells in gliomas and meningiomas. Brain Behav Immun.

[72] Zeng J, Li X, Sander M, Zhang H, Yan G, Lin Y (2021). Oncolytic viro-immunotherapy: an emerging option in the treatment of gliomas. Front Immunol..

[73] Zhang H, He J, Dai Z, Wang Z, Liang X, He F (2021). Pdia5 is correlated with immune infiltration and predicts poor prognosis in gliomas. Front Immunol..

[74] Lu T, Zheng Y, Gong X, Lv Q, Chen J, Tu Z (2021). High expression of hyaluronan-mediated motility receptor predicts adverse outcomes: a potential therapeutic target for head and neck squamous cell carcinoma. Front Oncol..

[75] He Z, Mei L, Connell M, Maxwell CA. Hyaluronan mediated motility receptor (hmmr) encodes an evolutionarily conserved homeostasis, mitosis, and meiosis regulator rather than a hyaluronan receptor. Cells-Basel. 2020;9. 10.3390/cells9040819 10.3390/cells9040819 PMC7226759 32231069

[76] Maxwell CA, Keats JJ, Belch AR, Pilarski LM, Reiman T (2005). Receptor for hyaluronan-mediated motility correlates with centrosome abnormalities in multiple myeloma and maintains mitotic integrity. Cancer Res.

[77] Kouvidi K, Nikitovic D, Berdiaki A, Tzanakakis GN (2014). Hyaluronan/rhamm interactions in mesenchymal tumor pathogenesis: role of growth factors. Adv Cancer Res.

[78] Uhlin U, Eklund H (1994). Structure of ribonucleotide reductase protein r1. Nature.

[79] Pontarin G, Ferraro P, Bee L, Reichard P, Bianchi V (2012). Mammalian ribonucleotide reductase subunit p53r2 is required for mitochondrial dna replication and dna repair in quiescent cells. Proc Natl Acad Sci USA.

[80] Zhan Y, Jiang L, Jin X, Ying S, Wu Z, Wang L (2021). Inhibiting rrm2 to enhance the anticancer activity of chemotherapy. Biomed Pharmacother..

[81] Aye Y, Li M, Long MJ, Weiss RS (2015). Ribonucleotide reductase and cancer: biological mechanisms and targeted therapies. Oncogene.

[82] Jin CY, Du L, Nuerlan AH, Wang XL, Yang YW, Guo R (2020). High expression of rrm2 as an independent predictive factor of poor prognosis in patients with lung adenocarcinoma. Aging (Albany NY).

[83] Gandhi M, Gross M, Holler JM, Coggins SA, Patil N, Leupold JH (2020). The lncrna lincnmr regulates nucleotide metabolism via a ybx1 - rrm2 axis in cancer. Nat Commun.

[84] Xie Y, Xue C, Guo S, Yang L (2021). Microrna-520a suppresses pathogenesis and progression of non-small-cell lung cancer through targeting the rrm2/wnt axis. Anal Cell Pathol (Amst).

[85] Li C, Zheng J, Chen S, Huang B, Li G, Feng Z (2018). Rrm2 promotes the progression of human glioblastoma. J Cell Physiol.

[86] Rasmussen RD, Gajjar MK, Tuckova L, Jensen KE, Maya-Mendoza A, Holst CB (2018). Author correction: brca1-regulated rrm2 expression protects glioblastoma cells from endogenous replication stress and promotes tumorigenicity. Nat Commun.

[87] Hill M, Tran N. Mirna interplay: mechanisms and consequences in cancer. Dis Model Mech. 2021;14. 10.1242/dmm.047662 10.1242/dmm.047662 PMC8077553 33973623

[88] Ali SZ, Langden S, Munkhzul C, Lee M, Song SJ. Regulatory mechanism of microrna expression in cancer. Int J Mol Sci. 2020;21. 10.3390/ijms21051723 10.3390/ijms21051723 PMC7084905 32138313

[89] Yang HD, Kim HS, Kim SY, Na MJ, Yang G, Eun JW (2019). Hdac6 suppresses let-7i-5p to elicit tsp1/cd47-mediated anti-tumorigenesis and phagocytosis of hepatocellular carcinoma. Hepatology.

[90] Liu Y, Hu X, Hu L, Xu C, Liang X (2021). Let-7i-5p enhances cell proliferation, migration and invasion of ccrcc by targeting habp4. Bmc Urol.

[91] Sun X, Xue H, Xiong Y, Yu R, Gao X, Qian M (2019). Gale promotes the proliferation and migration of glioblastoma cells and is regulated by mir-let-7i-5p. Cancer Manag Res.

[92] Georgiadou D, Boussata S, Keijser R, Janssen D, Afink GB, van Dijk M (2021). Knockdown of splicing complex protein pcbp2 reduces extravillous trophoblast differentiation through transcript switching. Front Cell Dev Biol..

[93] van Dijk M, Visser A, Buabeng KM, Poutsma A, van der Schors RC, Oudejans CB (2015). Mutations within the linc-hellp non-coding rna differentially bind ribosomal and rna splicing complexes and negatively affect trophoblast differentiation. Hum Mol Genet.

