# Supplementary material for: HELLPAR/RRM2 axis related to HMMR as novel prognostic biomarker in gliomas
Source: BMC Cancer. 2023 Feb 7;23:125. doi: 10.1186/s12885-023-10596-w (PMC9903609; doi:10.1186/s12885-023-10596-w)
Supplement: Supplementary file 1 — Additional file 1: Table S1. Univariate and multivariate analysis of demographic and clinical characteristics that correlate with OS of gliomas patients (CGGA database). Table S2. DEmRNAs in high and low HMMR expression gliomas samples (TCGA database). Table S3. Functional enrichment analysis for DEmRNAs based on HMMR expression. Table S4. GSEA analysis associated with HMMR. Table S5. DEmRNAs in GSE4290. Table S6. overlapped DEmRNAs. Table S7. Enrichment analysis of 41 DEmRNAs. Table S8. DEmRNAs-miRNA analysis. Table S9. DEmiRNAs in high and low HMMR expression gliomas samples (TCGA database). Table S10. DElncRNAs in high and low HMMR expression gliomas samples (TCGA database). Table S11. Univariate and multivariate analysis of demographic and clinical characteristics that correlate with OS of gliomas patients (TGGA database). Table S12. Correlation analysis of RRM2 (TCGA database). Table S13. Enrichment analysis of the correlated genes (correlation coefficient over 0.8) of RRM2 in gliomas. Table S14. Univariate and multivariate analysis of demographic and clinical characteristics that correlate with OS of gliomas patients (TGGA database). [file 12885_2023_10596_MOESM1_ESM.zip › Table S11 RRM2 Cox.docx]

| **Table S11. Univariate and multivariate analysis of demographic and clinical characteristics that correlate with OS of gliomas patients (TGGA database)** | | | | | | |
| --- | --- | --- | --- | --- | --- | --- |
| Characteristics | Total(N) | Univariate analysis | |  | Multivariate analysis | |
|  |  | Hazard ratio (95% CI) | P value |  | Hazard ratio (95% CI) | P value |
| WHO grade | 634 |  |  |  |  |  |
| G2 | 223 | Reference |  |  |  |  |
| G3 | 243 | 2.999 (2.007-4.480) | **<0.001** |  | 1.736 (1.115-2.704) | **0.015** |
| G4 | 168 | 18.615 (12.460-27.812) | **<0.001** |  | 3.901 (2.244-6.784) | **<0.001** |
| IDH status | 685 |  |  |  |  |  |
| Mut | 439 | Reference |  |  |  |  |
| WT | 246 | 8.551 (6.558-11.150) | **<0.001** |  | 3.205 (2.132-4.819) | **<0.001** |
| 1p/19q codeletion | 688 |  |  |  |  |  |
| codel | 170 | Reference |  |  |  |  |
| non-codel | 518 | 4.428 (2.885-6.799) | **<0.001** |  | 1.421 (0.859-2.351) | 0.171 |
| Age | 695 |  |  |  |  |  |
| <=60 | 552 | Reference |  |  |  |  |
| >60 | 143 | 4.668 (3.598-6.056) | **<0.001** |  | 1.514 (1.112-2.060) | **0.008** |
| Gender | 695 |  |  |  |  |  |
| Female | 297 | Reference |  |  |  |  |
| Male | 398 | 1.262 (0.988-1.610) | 0.062 |  | 1.225 (0.932-1.609) | 0.145 |
| RRM2 | 695 |  |  |  |  |  |
| Low | 348 | Reference |  |  |  |  |
| High | 347 | 4.441 (3.342-5.903) | **<0.001** |  | 1.553 (1.058-2.281) | **0.025** |
