# Supplementary material for: HELLPAR/RRM2 axis related to HMMR as novel prognostic biomarker in gliomas
Source: BMC Cancer. 2023 Feb 7;23:125. doi: 10.1186/s12885-023-10596-w (PMC9903609; doi:10.1186/s12885-023-10596-w)
Supplement: Supplementary file 2 — Additional file 2: Figure S1. The association of RRM2 expression with WHO grade, IDH statu, 1p/19q codeletion, and age in gliomas from TCGA database (A). Kaplan-Meier survival curve analysis and time-dependent ROC analysis showed that high RRM2 expression correlated to poor prognosis of gliomas patients from TCGA database (B). Figure S2. Correlation analysis of the ceRNA network. [file 12885_2023_10596_MOESM2_ESM.docx]

**
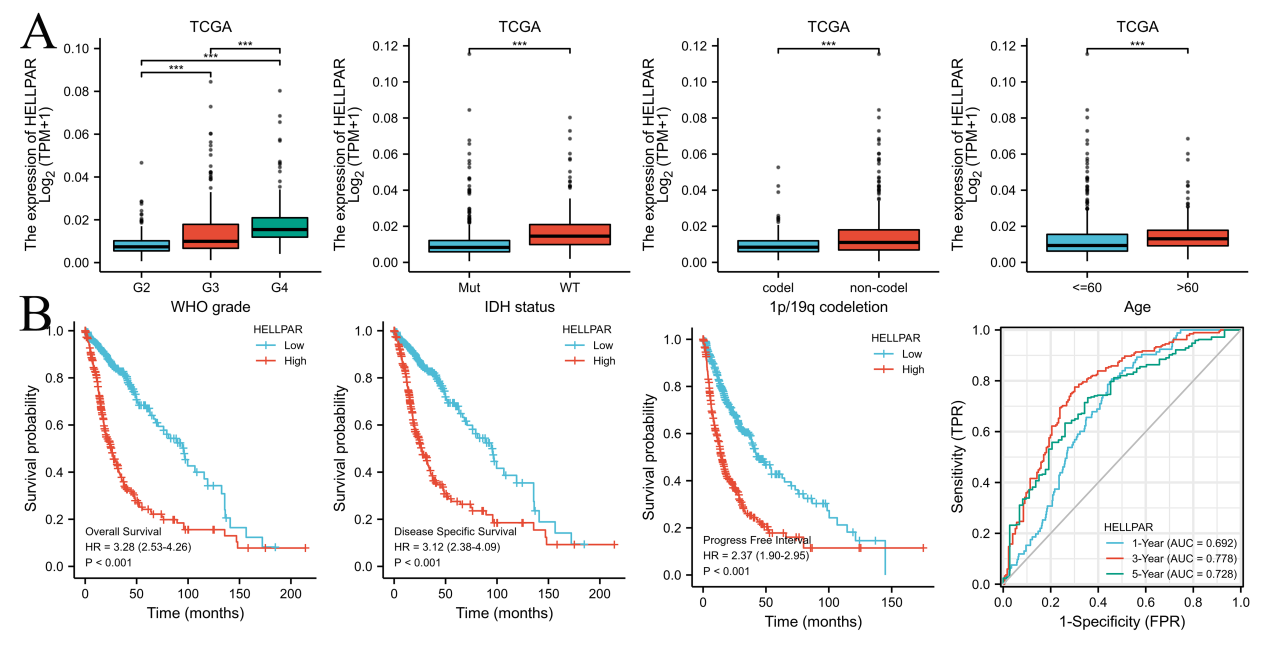
Figure S1.** The association of RRM2 expression with WHO grade, IDH statu, 1p/19q codeletion, and age in gliomas from TCGA database **(A)**. Kaplan-Meier survival curve analysis and time-dependent ROC analysis showed that high RRM2 expression correlated to poor prognosis of gliomas patients from TCGA database **(B)**.


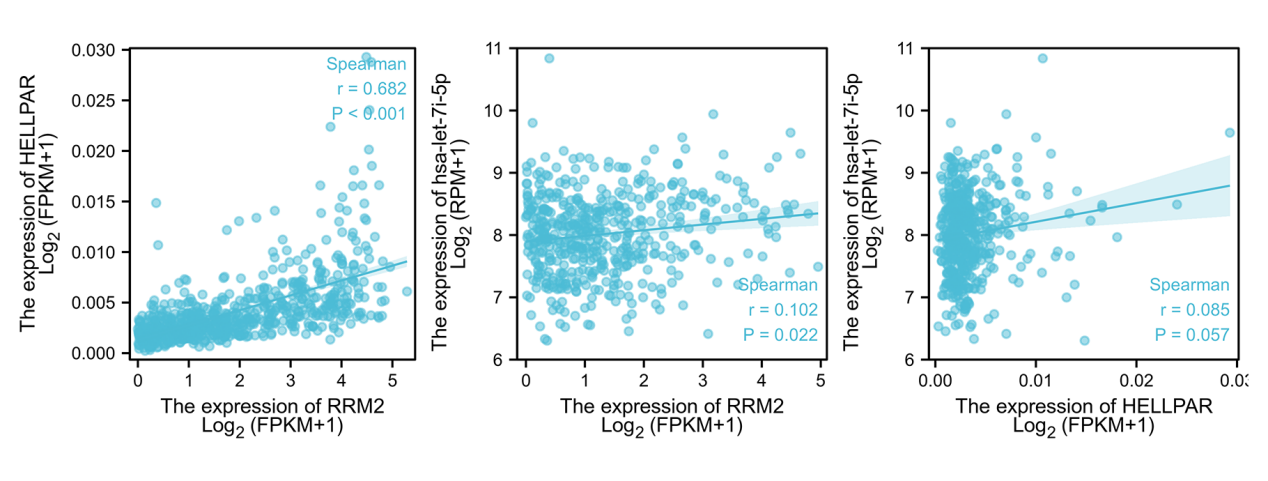


**Figure S2.** Correlation analysis of the ceRNA network.
